# Modelling enablers and barriers to improve uptake of a fruit and vegetable voucher scheme (the fresh street community) in England: a TISM-MICMAC approach

**DOI:** 10.1186/s12889-026-27063-3

**Published:** 2026-03-21

**Authors:** Jiang Pan, Clare Relton, Lisa Howard, Aira Ong Pan, Louise Hunt, Paridhi Garg, Jane Bradbeer, Manik Puranik, Rachel Sutton, Carol Wagstaff, Clare Pettinger

**Affiliations:** 1 Faculty of Health, University of Plymouth, InterCity Place, Plymouth Railway Station, North Road East, Plymouth, PL4 6AB UK; 2 Department of Food and Nutritional Sciences, University of Reading, Reading, UK; 3 Queen Mary University of London, London, UK

**Keywords:** Health equity, Community-based intervention, Systems thinking, Food access, Public health nutrition, Participatory modelling, Fruit and vegetable voucher scheme, United Kingdom

## Abstract

**Background:**

Improving equitable access to fresh fruit and vegetables (FV) remains a critical public health priority in the UK, particularly in low-income communities where affordability, accessibility, and social barriers limit healthy food choices. The *Fresh Street Community* scheme, a place-based FV voucher initiative in England, aims to enhance dietary equity by enabling households to purchase fresh produce from local community centres. Building on prior feasibility research, this study applies a systems-modelling approach to explore how multiple enablers and barriers interact to influence scheme uptake and sustainability.

**Methods:**

Using Total Interpretive Structural Modelling (TISM) combined with MICMAC analysis, we engaged research team members, local household participants, and community food researchers across two contrasting sites in England – a coastal and an urban community. Through participatory modelling, factors identified from previous qualitative work were hierarchically structured and classified according to their driving and dependency power to identify key leverage points for improving intervention design and implementation.

**Results:**

The analysis revealed complex, multi-level interactions shaping scheme participation. Foundational structural drivers, such as convenient location, produce quality, and operational reliability, strongly influenced engagement. These interacted with social capital factors, including community relationships, peer advocacy, and local trust, which amplified awareness and reduced stigma. Resource adequacy emerged as a pivotal linkage factor, determining the stability and reach of the intervention.

**Conclusions:**

Effective and sustainable FV voucher schemes require coordinated, multi-level strategies that strengthen physical and economic access, mobilise community networks, and secure sufficient resources. This systems-modelling approach offers policymakers and future researchers a practical framework to address persistent challenges in food access and health equity, supporting the development of more resilient, impactful food intervention schemes.

**Supplementary Information:**

The online version contains supplementary material available at 10.1186/s12889-026-27063-3.

## Introduction

Ensuring equitable access to fresh fruits and vegetables (FV) remains a persistent public health priority in the UK, especially in socio-economically deprived communities where affordability and accessibility are key barriers to healthy eating [1, 2]. Offering price discounts or subsidies on healthy foods increases healthy food purchasing [3] and consumption [4], especially when access to FVs is also improved [5]. Hence, over the years, many food and nutrition interventions have been implemented to encourage FV consumption in the UK [6–10].

Fresh Street is one such intervention. It is a place-based approach where paper FV vouchers are offered to all households regardless of household type, size, or income [6] for fresh FV supplied by local independent (non-supermarket) suppliers [2, 11]. Fresh Street Community (i.e., FoodSEqual-Health project) embeds this approach within community hubs, thus enabling households to use vouchers to purchase FV from community centres. The successful implementation and uptake of such schemes depend on a complex interplay of different types of factors that must be carefully understood and addressed to maximise program effectiveness.

Building on the recent Fresh Street Community feasibility study “*Exploring enablers and barriers to a fruit and vegetable voucher scheme in England*,” which identified a broad range of factors that influence participant engagement and scheme success [12], this study seeks to develop a structured, systems-level understanding of how these factors interrelate and influence one another. These factors range from individual-level considerations such as awareness, accessibility, and personal preferences to broader structural and organisational issues including program design, community infrastructure, and organisational capacity [12]. While these findings provide valuable insights into the multifaceted nature of voucher scheme implementation, they also highlight the need for a more structured understanding of how these various factors relate to and influence one another within the broader system (i.e., the network of interacting factors that shape uptake and use of the Fresh Street Community voucher scheme).

Understanding the hierarchical relationships and leverage points among enablers and barriers is critical for three reasons: (1) it shifts the focus from isolated interventions to systems-level approaches that address interconnected factors; (2) it identifies which factors exert the greatest influence on uptake, enabling policymakers and practitioners to priorities limited resources strategically; and (3) it provides evidence-based guidance for designing and scaling similar food access interventions across disadvantaged communities, with direct implications for reducing health inequities.

To extend this foundational work, the present study employs Total Interpretive Structural Modelling (TISM) to hierarchically map complex interrelationships among these factors based on expert and stakeholder input [13]. Concurrently, Matrix Impact Cross Multiplication Applied to Classification (MICMAC) analysis classifies factors by their driving and dependence power, identifying critical leverage points within the system [14]. Together, these methods provide a comprehensive framework to elucidate which factors exert the greatest influence on Fresh Street Community scheme uptake.

Specifically, the study seeks to:

- Identify and map the hierarchical relationships among the factors previously identified in the Fresh Street Community feasibility study.
- Classify these factors based on their driving and dependent power to reveal leverage points for practical implementation and resource allocation.
- Generate evidence-based implementation strategies for scaling and sustaining voucher schemes in low-income communities across the UK, with broader applicability to other food access interventions.

## Materials and methods

### Study design and theoretical framework

This study builds on the previously conducted exploratory qualitative research into the enablers and barriers of the Fresh Street Community voucher scheme [12]. A cross-sectional, participatory modelling approach was adopted using TISM to determine hierarchical relationships and MICMAC analysis to assess the influence and dependence of each factor.

TISM and MICMAC are systems analysis techniques that structure complex, multi-factor problems into hierarchical models by identifying causal relationships and classifying factors by their driving (influencing) and dependence (being influenced) power [15]. Originally developed in operations management and organisational research, they have increasingly been applied to healthcare challenges, including healthcare waste management, Health 4.0 implementation barriers, medical oxygen management, medical tourism development, commercialisation of healthcare innovations, and COVID-19 vaccination programmes [14, 16–19]. Their application to community-based healthy food interventions, however, remains relatively new.

TISM methodology was selected for its capacity to transform unclear and poorly articulated relationships into clear, well-designed theoretical models that establish causal relationships among factors in a structured form [13, 14, 17, 18, 20]. MICMAC analysis is integrated with TISM to further classify factors by their driving and dependence power [13, 16, 21]. The TISM-MICMAC approach involves both qualitative and quantitative logic [13, 14]. Qualitative when the variables are compared in pairs by taking expert opinion and quantitative when it comes to transitivity and reachability matrix check [22].

### Intervention setting

“Fresh Street Community” was implemented in two areas of high material deprivation in the UK [23]. Details of the main intervention’s recruitment and sampling strategy have been published separately [2, 11, 24], and the main evaluation of Fresh Street was published in the *Philosophical Transactions of the Royal Society B* in 2025 [25]. A summary of the intervention is outlined below for context.

- Southwest England Coastal implementation site (Whitleigh, Plymouth).

From January to September 2024, £10 vouchers were distributed fortnightly via doorstep drops on the intervention street. On a fortnightly basis, orders could be placed on Tuesdays from 10 am to 12 pm at a local community venue, with collections on Thursdays of the same week at the same location. Pre-packed locally grown and supplied seasonal FV bags delivered by a regional FV wholesaler, each worth £5, were prepared for collection. Additionally, since April 2024, a market stall was held in a prominent place in the community, allowing people to choose FV produce, using either vouchers, cash or card.

- Central England implementation site (Whitley, Reading).

From November 2023 to June 2024 (Stage 1), £10 vouchers were distributed fortnightly via doorstep delivery on the intervention street. In Stage 2, June 2024, delayed intervention street (i.e., households on this street were the control group, receiving only project information letters without vouchers in Stage 1) was added. In Stage 3, July 2024, doorstep delivery ceases and vouchers were collected by households from a local community hub, and their value increased to £10 weekly to address the rising cost of living. Vouchers could be collected on Thursdays, Fridays, and Saturdays from the hub, which also operated the Saturday morning FV market stall, which was stocked by a locally-based FV wholesaler.

At both sites, people could buy FV using vouchers, cash or card. Additionally, on some collection days, FV preparation and cooking engagement activities were organised [12, 25].

### Participant recruitment and sampling

Participants were purposively sampled from the prior enablers and barriers qualitative study [12], which engaged approximately 120 households per site through observations, door-knocking, informal conversations, and interviews. Insights from that broader engagement were documented, shared, and discussed in regular meetings between the research team and community food researchers, and they informed both factor selection and interpretation in the present study. Inclusion criteria for eligible participants were 18 years old and above who must have had operational or lived experience with the voucher scheme across either of the two implementation sites [11]. Per site, representation from each of the three core stakeholder groups were included: (1) research team members, (2) community food researchers (CFRs) and (3) households (i.e., community participants in the scheme). This ensured deep contextual familiarity [26], with the site-specific voucher scheme’s operation, challenges, and opportunities, providing the foundation knowledge necessary for meaningful TISM-MICMAC analysis [16, 27].

The multi-stakeholder composition of the sample reflects the recognition that different participant groups bring distinct perspectives and expertise to understanding voucher scheme dynamics. Research teams in each site consisted of academic project coordinators, research fellows, and logistics managers. Their responsibilities included conducting door-to-door voucher drops, conversations, collecting FV orders from participants, placing orders with the suppliers, setting up venues, designing and conducting engagement activities, developing research plans, recording interviews, and taking observation notes. They had a deep understanding of the study’s objectives, methodologies, and challenges. Their participation provided valuable insights based on their experience of implementing Fresh Street Community [28]. CFRs are members of the local communities we worked in and who were recruited and trained to actively engage in the research activities. Their responsibility included door-to-door conversations, voucher drops, collecting FV orders from participants, setting up venues, designing and conducting engagement activities, contributing to research design, engaging with local residents through casual conversations or interviews, and taking observation notes. They bring valuable local knowledge, lived experiences, and cultural understanding to the research process, helping ensure that the study is relevant, accessible, and meaningful to the community [29, 30]. Community participants are the end-users and beneficiaries of the voucher scheme, who are directly impacted by the intervention. They provide lived experience and user-centred viewpoints essential for comprehensive system understanding.

FV Vendors were excluded from this phase as their role centred on back-end supply logistics rather than frontline operations affecting household uptake (e.g., distribution, community engagement). Their perspectives were deemed less relevant to modelling factor interrelationships.

For each site, all core members responsible for the daily operation of the scheme were invited. Only one research team member and one community food researcher were unable to participate due to workload and availability constraints. A total of 10 participants across each site took part in the study. There were five research team members (Plymouth *n* = 2, Reading *n* = 3), three CFRs (Plymouth *n* = 2, Reading = 1) and two community participants (Plymouth *n* = 1, Reading *n* = 1). The two community participants had long-term engagement with the scheme, attended regularly, and one also helped with delivery on a voluntary basis. Recruitment of additional household participants proved challenging because many eligible households were unable or unwilling to commit to the three‑hour data collection period.

### Data collection

Selected participants received a paper or digital invitation letter, information sheet, and consent form. Data collection was conducted through structured TISM questionnaires administered between October 15th 2024, and February 28th 2025, providing an extended timeframe that accommodated participant schedules while ensuring thoughtful completion of the complex relationship assessments required for the analysis.

#### Training and preparation procedures

Prior to data collection, participants received training on TISM-MICMAC methodology and questionnaire completion procedures. While previous TISM-MICMAC studies [14, 16, 31] rely heavily on expert intuition to assess factor relationships, variability in stakeholders’ interpretive frameworks are often overlooked, particularly in multi-stakeholder contexts like community health interventions [2]. The design and inclusion of participant training in this study ensured shared understanding of factors and methodologies, minimising response variability, and enhancing validity of relationship assessments.

Training was delivered either in a small-group or individual online sessions, depending on participant availability and preference. The training protocol was designed to ensure consistent understanding of both the technical aspects of the TISM–MICMAC method and the contextual meaning of each factor under examination [12] (See Table S1).

Participants were also introduced to the logic of pairwise comparison, a core element of TISM-MICMAC methodology, where they were asked to determine whether a given factor directly influences another [32]. The training incorporated practice exercises using sample questions that allowed participants to become familiar with the response format and reasoning processes required for effective TISM data provision.

#### TISM questionnaire

The TISM questionnaire was site-specifically tailored to account for specificity in validated factors between Whitleigh in Plymouth (23 factors) and Whitley in Reading (21 factors) [12]. The questionnaire design followed established TISM protocols that systematically examine pairwise relationships between all identified factors [13, 14]. For each site, the instrument included:

1. Factor glossary: a detailed list that defines each enabler and barrier in clear, consistent language so all participants understand exactly what each factor means in the context of the study.
2. Pairwise comparison: a systematic process where participants assess every possible pair of factors to determine whether one factor directly influences the other, typically by answering “Yes” or “No” to each comparison/question [15]. For example, “*Does fresh & long-lasting quality influence direct financial support?”*
3. Open-text field for brief causal explanations if answered “Yes”, expounding how/why there is a relationship.

Each participant independently assessed every possible factor pair. For each pair, they (1) identified whether one factor influences the other and in which direction, and (2) explained how and why that influence occurs [33]. Yes, is used if factor A influences B, and No if A does not influence B. Entry for a particular interaction was given if more than half of the participants agreed (See Table S2).

### Data analysis processes

**Fig. 1:**
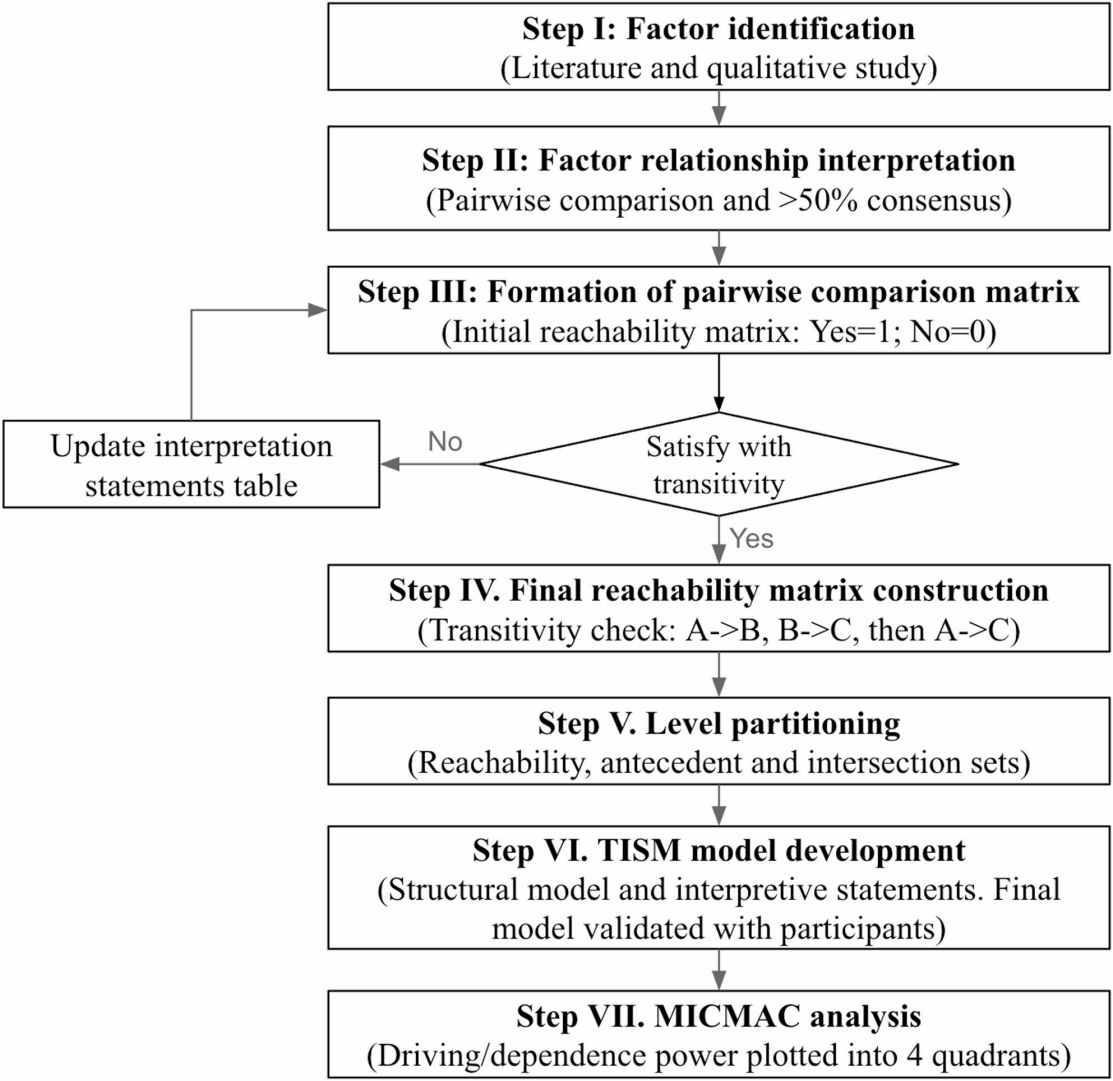
Overview of the TISM–MICMAC analysis procedure

TISM-MICMAC analysis procedure systematically followed established protocols [14, 32], visualised in Fig. 1 with the following steps:

#### Step I. factor identification

Factors (enablers and barriers) were identified based on the previous qualitative study and literature review across Plymouth (23 factors) and Reading (21 factors) [12].

#### Step II. factor relationship interpretation

The next step determines whether *factor A will influence or enhance factor B* but also extracts an explanation of *how* they influence or enhance each other. Each factor was compared against all other factors, where the ith element was individually compared with all elements from the (i + 1)th to the nth element [32]. From the 23 identified factors in Plymouth, a total of 506 comparisons were made. This is calculated using the formula n × (*n* − 1) = (23 × 22 = 506). Reading then had 420 pairwise items. Each pairwise comparison was assessed by the participants. If > 50% of the experts approved the influential relationship between two factors, it was marked as ‘“Yes”; otherwise, it was marked as ‘“No” [14].

#### Step III. formation of pairwise comparison matrix

The initial reachability matrix is formed by plotting “Yes” as ‘1’ and “No” by ‘0’ (see Appendix A: Table A1 & A2 in Supplementary Material).

#### Step IV. final reachability matrix construction

A further transitivity check is conducted to ensure the logical consistency of these relationships. The transitivity rule states that if factor A relates to factor B, and factor B relates to factor C, then factor A must also relate to factor C. As a result, the final reachability matrix is created (see Appendix B: Table B1 & B2 in Supplementary Material), where “1*” is used to denote a transitive relationship.

#### Step V. level partitioning

The final reachability matrix was used to determine the hierarchical levels of each factor by determining the reachable sets, antecedent sets, and intersection sets. The reachable set comprises all elements present in the row corresponding to each factor, while the factors present in the column represent the antecedent set. The intersection set includes factors common to both the reachable and antecedent sets. Factors for which the reachable and intersection sets are identical occupy the first level in the hierarchy. These factors are then removed from consideration in the next round of levelling. This process continues iteratively, removing factors with matching reachable and intersection sets at each step until a full hierarchy is established. A total of eight levels were identified in Plymouth, while six in Reading. The combined levels of factors for each site are presented in Appendix C: Table C1 & C2 in Supplementary Material.

#### Step VI. TISM model development

**Fig. 2:**
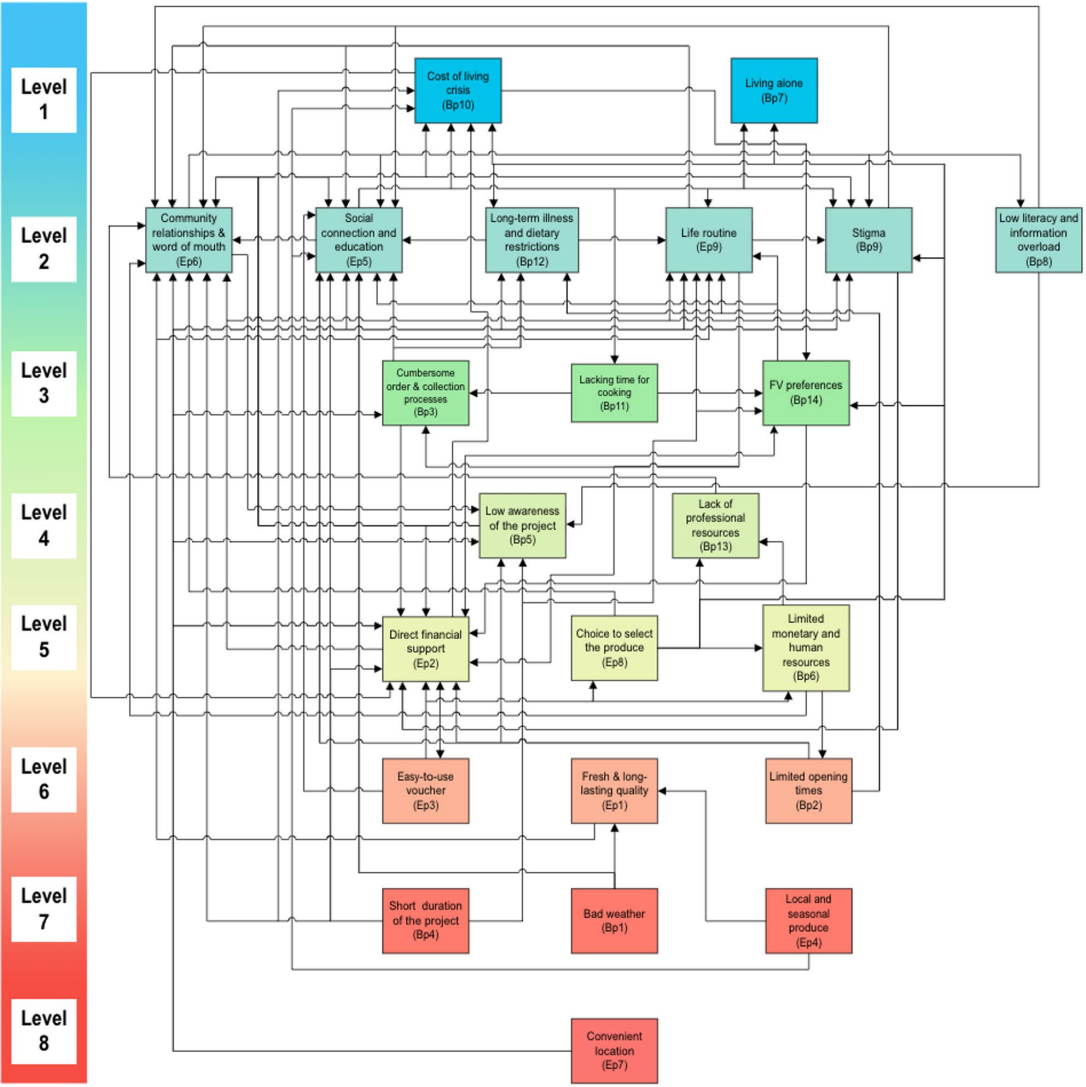
TISM model of Plymouth. ***Note***: Nodes represent factors; directed arrows indicate influence pathways. *Colour coding*: red (Levels 7–8) = foundational drivers (high driving power); yellow/green (Levels 3–6) = intermediate linkage factors; blue (Levels 1–2) = dependent outcomes (high dependence, low driving power). *Abbreviations: Ep* Enabler, Plymouth, *Bp* Barrier, Plymouth. Factor definitions in Table S1

The TISM model, as shown in Figs. 2 and 4 in Sect.  3, is a structural model that is developed based on level partition (Appendix C in Supplementary Material). Factors positioned at the first level of the hierarchy appear at the top, followed by factors from subsequent levels in descending order. The nodes represent the factors, and the links indicate the interpreted relationships among them. The model is complemented by compiled interpretive statements of each significant relationship (see Appendix D in Supplementary Material). Then, further assessment was done to enhance the model by eliminating insignificant relationships and retaining only those direct and transitive relationships whose interpretations are considered crucial. Insignificant relationships were identified by reviewing participants’ interpretive statements; only links for which participants articulated a clear, plausible, and direct causal mechanism relevant to scheme uptake were retained. Links lacking convincing interpretive justification, or judged to be trivial or overly indirect, were removed so that the final model contained only those direct and transitive relationships whose interpretations were considered crucial. The final TISM model was then taken back to participants for member checking, and most participants (8 out of 10) confirmed that the structure and retained relationships accurately reflected their understanding of how the scheme operates.

#### Step VII. MICMAC analysis

**Fig. 3:**
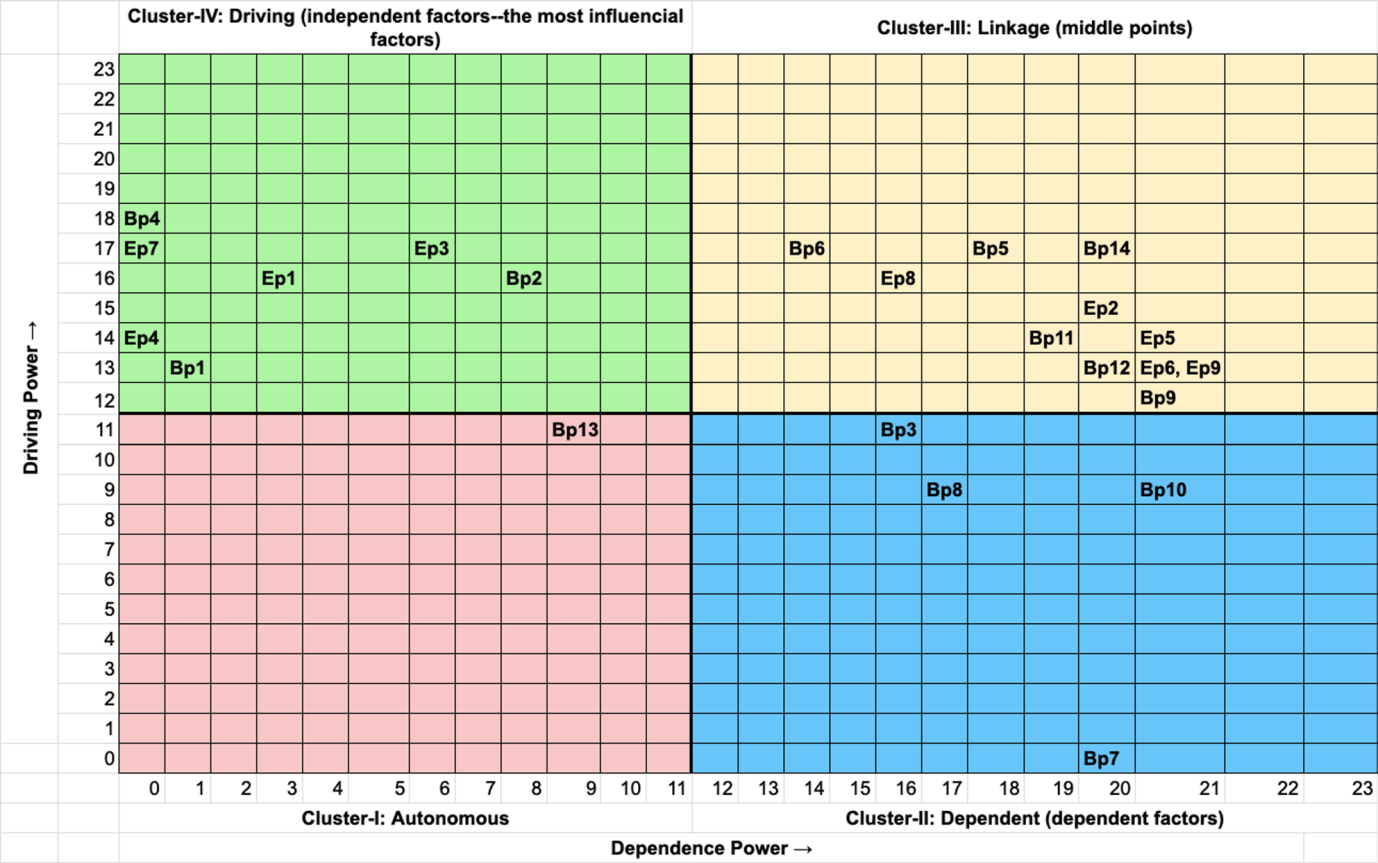
MICMAC diagram of Plymouth. Scatter plot of driving power (x-axis) versus dependence power (y-axis). *Clusters*: I = Autonomous (low driving/low dependence); II = Dependent (low driving/high dependence); III = Linkage (high driving/ high dependence); IV = Driving (high driving/low dependence). *Abbreviations: Ep* Enabler, Plymouth; *Bp* Barrier, Plymouth. Factor definitions in Table S1

The binary matrix (see Appendix E: Table E1 & E2 in Supplementary Material) was prepared from the final reachability matrix by placing zero in the diagonal elements. The driving power and dependence of each factor were calculated by summing the rows and columns, respectively. Then, they are plotted according to their driving and dependence powers categorised into four clusters: autonomous, dependent, linkage, and driving factors, as shown in Figs. 3 and 5 in Sect.  3.

All steps of the TISM-MICMAC process were conducted using spreadsheet and visualisation software (Microsoft Excel and Microsoft Visio), and TISM calculations and MICMAC plotting are presented in the results section below.

## Results

TISM and MICMAC results are presented per site in the following sections.

### TISM results for plymouth site

The TISM model resulted in an eight-level hierarchical structure (Fig. 2), mapping 87 interpretive relationships and the flow of influence among 23 identified enablers and barriers. The hierarchy is organised such that foundational factors at Level 8 (shown in red) exert the strongest influence on other factors, while factors toward Level 1 (shown in blue) have decreasing direct influence with minimal driving power. For example, Ep7 (Convenient location, Level 8) and Bp4 (Short duration, Level 7) drive intermediate factors (Levels 3–6), which shape dependent outcomes at Levels 1–2 including Bp10 (Cost of living crisis) and Bp7 (Living alone). The colour gradient from red (Level 7–8 as foundational factors) through yellow and green (Levels 3–6 as intermediate factors) to blue (Level 1–2 as dependent factors) visually reinforces this organisation, with most influential factors in warm colours and least influential factors in cool colours. The specific meaning of each link is detailed in Appendix D: Table D1 in Supplementary Material.

### MICMAC results for Plymouth site

The MICMAC analysis classified the 23 identified factors into four distinct clusters based on their driving power and dependence power (Fig. 3).

#### Cluster I: autonomous factors

This cluster contained only one factor: Bp13 (Lack of professional resources). This means that this factor had minimal influence on the system and was also relatively unaffected by other factors within the model for Plymouth.

#### Cluster II: dependent factors

This cluster comprised four factors which are highly dependent on others but exerting little driving influence themselves: Bp3 (Cumbersome order and collection processes), Bp7 (Living alone), Bp8 (Low literacy and information overload), Bp10 (Cost of living crisis).

#### Cluster III: linkage factors

This critical cluster contained factors with significant interconnectedness, acting as both drivers and dependents within the system. Factors includes: Bp5 (Low awareness of the project), Bp6 (Limited monetary and human resources), Bp9 (Stigma), Bp11 (Lacking time for cooking), Bp12 (Long-term illness and dietary restrictions), Bp14 (FV preferences), Ep2 (Direct financial support), Ep5 (Social connection and education), Ep6 (Community relationships & word of mouth), Ep8 (Choice to select the produce), and Ep9 (Life routine).

#### Cluster IV: driving factors

This cluster contained the most influential factors, exerting strong driving power on the system while being relatively independent: Bp1 (Bad weather), Bp2 (Limited opening times), Bp4 (Short duration of the project), Ep1 (Fresh & long-lasting quality), Ep3 (Easy-to-use voucher), Ep4 (Local and seasonal produce), Ep7 (Convenient location).

### TISM results for reading site

**Fig. 4:**
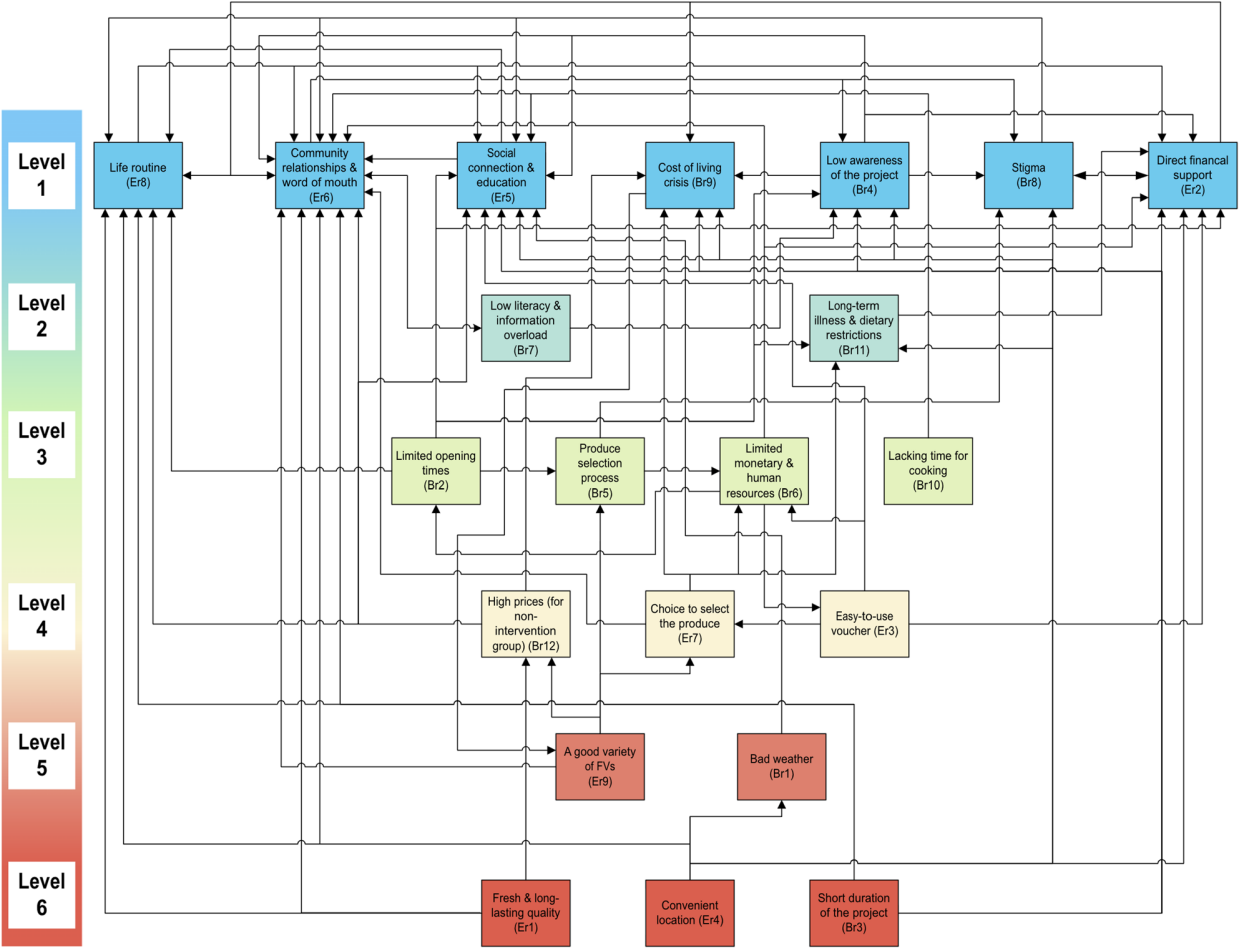
TISM model of Reading. Nodes represent factors; directed arrows indicate influence pathways. *Colour coding*: red (Levels 7–8) = foundational drivers (high driving power); yellow/green (Levels 3–6) = intermediate linkage factors; blue (Levels 1–2) = dependent outcomes (high dependence, low driving power). *Abbreviations*: Er = Enabler, Reading; Br = Barrier, Reading. Factor definitions in Table S1

The TISM model resulted in a six-level hierarchical structure (Fig. 4), revealing 74 interpretive relationships and the flow of influence among 21 identified enablers and barriers. The hierarchy is organised such that foundational factors at Level 6 (shown in red) exert the strongest influence on other factors, while factors toward Level 1 (shown in blue) have decreasing direct influence with minimal driving power. For example, Er1 (Fresh & long-lasting quality, Level 6), Er4 (Convenient location, Level 6), and Br3 (Short duration of the project, Level 6) are foundational drivers that influence intermediate factors (Levels 3–5), which shape dependent outcomes at Levels 1–2 including Er2 (Direct financial support), Er5 (Social connection & education), Er6 (Community relationships & word of mouth), Er8 (Life routine), Br4 (Low awareness), Br8 (Stigma), and Br9 (Cost of living crisis). The colour gradient from red (Level 6 as foundational factors) through yellow and green (Levels 3–5 as intermediate factors) to blue (Levels 1–2 as dependent factors) visually reinforces this organisation, with most influential factors in warm colours and least influential factors in cool colours. The specific meaning of each link is detailed in Appendix D: Table D2 in Supplementary Material.

### MICMAC results for reading site

**Fig. 5:**
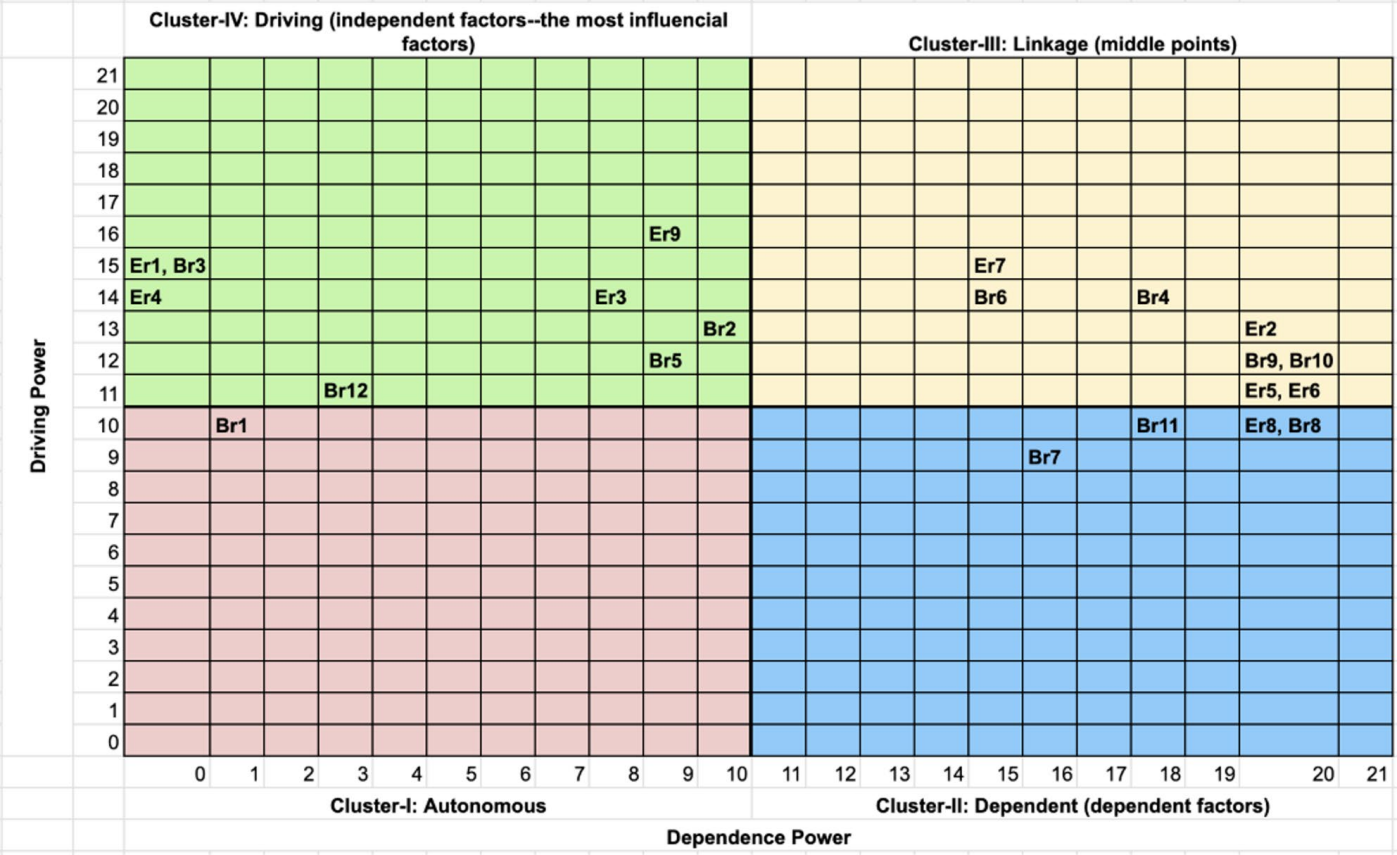
MICMAC diagram of Reading. Scatter plot of driving power (x-axis) versus dependence power (y-axis). *Clusters*: I = Autonomous (low driving/low dependence); II = Dependent (low driving/high dependence); III = Linkage (high driving/ high dependence); IV = Driving (high driving/low dependence). *Abbreviations*: Er = Enabler, Reading; Br = Barrier, Reading. Factor definitions in Table S1

The MICMAC analysis classified the 21 identified factors into four distinct clusters, Fig. 5, based on their driving power (influence on others) and dependence power (being influenced by others), revealing the system’s underlying architecture and intervention priorities.

#### Cluster I: autonomous factors

This cluster contained only one factor: Br1 (Bad weather). This means that this factor had minimal influence on the system and was also relatively unaffected by other factors within the model for Reading.

#### Cluster II: dependent factors

This cluster comprised four factors which are highly dependent on others but exerting little driving influence themselves: Br7 (Low literacy and information overload), Br11 (Long-term illness and dietary restrictions), Er8 (Life routine), Br8 (Stigma).

#### Cluster III: linkage factors

This critical cluster contained factors with significant interconnectedness, acting as both drivers and dependents within the system. Factors include: Er7 (Choice to select the produce), Br6 (Limited monetary and human resources), Br4 (Low awareness of the project), Er2 (Direct financial support), Br9 (Cost of living crisis), Br10 (Lacking time for cooking), Er5 (Social connection and education), and Er6 (Community relationships and word of mouth).

#### Cluster IV: driving factors

This cluster contained the most influential factors, exerting strong driving power on the system while being relatively independent: Er1 (Fresh and long-lasting quality), Br3 (Short duration of the project), Er4 (Convenient location), Br12 (High prices—for non-intervention group), Er3 (Easy-to-use voucher), Er9 (A good variety of FVs), Br5 (Produce selection process), and Br2 (Limited opening times).

## Discussion

### Key drivers and hierarchical influence

The hierarchical structures revealed through TISM analysis demonstrate that Fresh Street Community voucher scheme uptake is not simply a matter of individual choice or programme design, but rather emerges from a complex web of multilevel factors operating across individual, interpersonal, organisational, and structural levels [34]. This finding aligns with systems thinking approaches that recognise complex health challenges require comprehensive understanding of interconnected factors rather than linear cause-and-effect relationships [35, 36]. The eight-level hierarchy identified in Plymouth and six-level hierarchy in Reading illustrate how foundational drivers positioned at the base of the TISM models (levels 7–8 in Plymouth; Level 5–6 in Reading) exert causal influence on intermediate factors, which in turn shape dependent outcomes at the apex (levels 1–2), creating systematic patterns of influence that must be understood holistically.

In both sites, foundational drivers such as *Convenient location* (Ep7/Er4), *Fresh & long-lasting quality* (Ep1/Er1), and *Easy-to-use voucher* (Ep3/Er3) emerged as critical enablers, positioned at the lowest levels of the hierarchy. These factors exerted the strongest influence on the system while remaining relatively unaffected by other factors, suggesting their vital role in shaping participation and satisfaction. These findings align with food access literature emphasising that physical accessibility, procedural simplicity, and sensory appeal are key determinants of food access patterns, particularly among vulnerable populations with limited transportation options [37–40]. Similarly, *Short project duration* (Bp4/Br3) and *Bad weather* (Bp1/Br1) were strong barriers, reflecting how temporal constraints and environmental factors negatively impact intervention sustainability [41]. The consistency of these factors across both sites suggests they represent universal requirements for effective voucher scheme operation, regardless of local context. Strategically, these findings necessitate prioritising geospatial optimisation of locations to better serve disadvantaged communities [42], investing in cold-chain infrastructure to preserve produce quality [43], and designing flexible timelines to buffer against inconvenience and weather-related disruption [44].

The MICMAC analysis further revealed the roles of these factors by classifying them into autonomous, dependent, linkage, and driving clusters. Dependent factors such as *Living alone* (Bp7) and *Cost of living crisis* (Bp10/Br9) occupied the highest levels in the TISM hierarchies, indicating their susceptibility to influence from other factors in the system but limited capacity to drive change. They represent system outcomes that reflect the functioning of downstream drivers and linkage factors [17, 18]. While these factors may not be direct intervention targets, they are better viewed as system outcomes and may be valuable indicators of performance. Linkage factors demonstrated high interconnectedness, acting as both drivers and dependents within the system [14, 16]. These factors (e.g., *Social connection & education* (Ep5/Er5), *Community relationships* (Ep6/Er6), and *Resource availability* (Bp6/Br6)) in both sites emphasises the importance of addressing interconnected social and operational elements to foster sustainable engagement and generate cascading effect throughout the system.

### Multi-level intervention implications

The findings from the TISM-MICMAC analysis strongly support the need for multi-level, mutually reinforcing interventions to maximise the uptake and sustained impact of FV voucher schemes in disadvantaged communities. Rather than acting in isolation, interventions targeting structural, social, and resource-related determinants are deeply interconnected, collectively shaping community engagement and scheme effectiveness.

Structural drivers, such as convenient location, reliable operation, and product quality, emerged as key foundational influences in both sites, exerting effects deep into the system hierarchy. Improving geographic accessibility, for example, not only reduces practical barriers but also underpins the effectiveness of upstream factors, such as access to financial support, community engagement, social connection, and integration of the project into routine, by ensuring the physical presence and visibility of the scheme within the community [2, 4]. High quality, accessible venues create a baseline of trust and usability that enhances both awareness and participation, paving the way for wider social mobilisation [6, 8]. However, it is important to acknowledge that such venues are often in short supply in areas of deprivation – many community centres or suitable spaces have been sold off or are lacking when compared to more affluent areas. This considerable disparity in community infrastructure can limit both where and how voucher schemes can be implemented, and places additional strain on schemes attempting to operate in the communities most likely to benefit from them. Investment in site selection, infrastructure, and operational standards therefore, does more than simply increase access; they also help to normalise voucher schemes, which in turn can mitigate stigma and support the growth of community partnerships [45, 46]. Structural interventions thus provide the essential groundwork upon which broader engagement strategies can be constructed.

Building on a solid structural foundation, social capital interventions, such as improving community relationships, leveraging peer networks and investing in participatory activities are vital for increasing awareness, fostering trust, and overcoming psychosocial barriers [29, 47]. Linkage factors like social connection and community word of mouth transmit the effects of foundational drivers into sustained behavioural engagement and positive scheme experiences. For instance, expanding community food researcher models [29] and peer-led outreach (e.g., community champion visits, neighbour-to-neighbour introductions, shopping trips with existing participants, local resident-led market stalls, and door-to-door promotion) can reinforce existing networks, creating ripple effects that increase awareness, encourage new participants and reduce misconceptions or stigma surrounding voucher use [2, 48]. Importantly, social engagement interventions become far more effective when aligned with high-quality, well-located, and adequately resourced schemes highlighting the synergy between infrastructure and community mobilisation.

Resource adequacy was a linkage factor with wide‑reaching influence. Sufficient financial and human resources are the backbone of a reliable and sustainable voucher scheme, enabling long-term delivery and a lasting impact on the community’s healthy eating habits. Adequate resources resolve the operational and logistical issues essential for daily functioning. These include:

- Staffing limitations – having enough trained, motivated personnel or volunteers to cover opening hours, support participants, and maintain quality service.
- Operational reliability – ensuring voucher distribution, produce supply, and redemption processes work smoothly.
- Infrastructure readiness – adequate facilities, physical space, IT systems, and storage to deliver the programme efficiently.
- Financial stability – consistent funding to purchase fresh & good quality produce, cover staff wages, and meet running costs.

Resources act as catalysts for amplifying the benefits of both structural drivers (e.g., location, quality, reliability) and social capital strategies (e.g., relationship-building, trusted messengers). Adequate resources ensure that access points are open and welcoming, and enable network mobilisation without overburdening staff or volunteers. However, the rising cost of living in the UK has intensified these resource demands. To alleviate financial pressures on households, voucher values were increased in Reading, but this in turn heightened the project’s operational cost and stress. Moreover, the produce range had to be reduced because certain items became too expensive for many stall users. The cost of living crisis has also shifted participants’ food choices and cooking habits – many now turn to unhealthy, ultra-processed foods that are easy to find, frequently discounted, quick to prepare, and cheaper than cooking from scratch [49]. This shift is further reinforced by commercial food industry marketing, which strategically promotes ultra-processed products as convenient, affordable solutions during economic hardship, actively shaping consumer choice and food desirability away from fresh produce [50, 51]. These changes create significant challenges for achieving sustained improvements in dietary quality, even when structural and social supports are in place.

In summary, interventions must continue to adapt to broader economic fluctuations, ensuring resource adequacy and flexibility, maintaining variety and quality of produce, and supporting behavioural change. Only through integrated approaches that address structural, social, and resource barriers together can FV voucher schemes deliver long-term, equitable improvements in food access and health.

### Strengths and limitations of the study

This study is the first to apply TISM-MICMAC methodology to systematically model the complex dynamics influencing FV voucher scheme uptake in deprived communities. The hierarchical structures revealed at both sites extend understanding beyond financial considerations to encompass social, operational, and structural determinants.

The application of TISM-MICMAC methodology to community food interventions represents a significant methodological advancement in understanding complex health system dynamics [14, 16, 48]. Unlike traditional linear evaluation approaches, this participatory modelling technique captured the multidirectional relationships and feedback loops that characterise real-world implementation [31]. The training component implemented prior to data collection addresses a critical gap identified in previous TISM applications, where stakeholder interpretive frameworks were often overlooked. The provision of standardised training helped minimise response variability due to methodological unfamiliarity while maximising the validity of relationship assessments. Furthermore, although the TISM questionnaire was lengthy (approximately 3 h per participant), using a self-administered format and providing preparatory training helped to reduce participant burden and allowed respondents to complete the pairwise comparisons at their own pace. A key strength of this study is that it achieved near-complete participation among those most closely involved in the scheme’s design and delivery where only one research team member and one community food researcher were unable to take part due to workload and availability constraints.

The multi-stakeholder approach, incorporating research team members, community food researchers, and household participants, aligns with principles of community-based participatory research that emphasize the value of diverse perspectives in understanding complex systems [12, 52]. This methodological innovation is particularly relevant given the growing recognition that community food interventions operate within complex social-ecological systems that require deeper understanding of local context and stakeholder experiences.

However, the study’s reliance on purposive sampling from a previous qualitative study introduces potential limitations regarding generalisability [53]. While this approach ensured deep contextual familiarity, the findings may not capture the full spectrum of barriers and enablers that could emerge in different implementation contexts or with alternative stakeholder configurations [54]. The reliance on expert opinion and consensus-based relationship determination may introduce bias, particularly when stakeholder groups have divergent perspectives on system functioning. The cross-sectional nature of the analysis limits understanding of how relationships evolve over time and in response to changing circumstances. Furthermore, the locations of the sites might not represent other UK contexts.

Future research could consider longitudinal applications of systems modelling to capture evolving dynamics and intervention effects over time. Additionally, integration with quantitative outcome data may strengthen the evidence base by linking systems structures to measurable health and behavioural impacts. Moreover, to enhance the generalisability of findings related to fruit and vegetable voucher schemes, future research should adopt a broader scope by including more participants, diverse geographic locations and variations in voucher delivery methods. This expansion is essential to validate the findings across different socio-economic and cultural contexts, providing robust evidence for researchers, policymakers and practitioners [12].

## Conclusion

This study applied a TISM-MICMAC approach to map and classify the complex, multi-level factors influencing uptake of the Fresh Street Community FV voucher scheme. The analysis revealed that scheme participation is shaped by an interconnected hierarchy of structural drivers, social capital factors, and resource adequacy, with each domain exerting cross-cutting influence on accessibility, engagement, and sustainability.

Key structural drivers, such as convenient location, high produce quality, and reliable operations, formed the foundation on which other factors operated. These interacted closely with social capital elements, including trusted community relationships and local knowledge sharing, which were shown to amplify awareness and encourage sustained behavioural engagement. Resource adequacy emerged as a pivotal linkage factor, enabling not only the resolution of operational challenges but also the effectiveness of both structural and social interventions.

These insights underline the need for coordinated, multi-level strategies that address foundational access barriers, strengthen community networks, and ensure stable funding and human capacity. Targeting only one domain is unlikely to deliver lasting improvements; instead, progress at each level reinforces the others, creating a more resilient and responsive intervention system. As communities and policymakers seek to close persistent gaps in food access and health equity, adopting such approaches offers a promising pathway to designing, scaling, and sustaining community‑based nutrition interventions with lasting impact.

## Supplementary Information

Supplementary Material 1.

Supplementary Material 2.

Supplementary Material 3.

## Data Availability

The datasets used and analysed during the current study are available from the corresponding author on reasonable request.

## Abbreviations

CFR: Community Food Researcher
FV: Fruit and Vegetable
MICMAC: Matrix Impact Cross Multiplication Applied to Classification
TISM: Total Interpretive Structural Modelling
