# Supplementary material for: Modelling enablers and barriers to improve uptake of a fruit and vegetable voucher scheme (the fresh street community) in England: a TISM-MICMAC approach
Source: BMC Public Health. 2026 Mar 21;26:1399. doi: 10.1186/s12889-026-27063-3 (PMC13126962; doi:10.1186/s12889-026-27063-3)
Supplement: Supplementary file 1 — Supplementary Material 1. [file 12889_2026_27063_MOESM1_ESM.docx]

**Enablers & Barriers’ Explanation for Plymouth & Reading**

| **Plymouth** | |
| --- | --- |
| **Enablers** | *Explanation* |
| (Ep1) Fresh & long-lasting quality | Our produce quality and size are generally better than those from supermarkets. |
| (Ep2) Direct financial support | It’s free and provides direct financial support. It also gives people the opportunity to try new fruits and vegetables (FV) they haven't had before, without the risk of spending money. |
| (Ep3) Easy to use voucher | The voucher is easy for participants to use; the research team scans it to help people order produce. So community people have more time to interact with each other or research team. But it increases the workload for our research team. |
| (Ep4) Local & seasonal produce | All the produce we supply are seasonal and from local farmers. |
| (Ep5) Social connection & education | We set up friendly environment (e.g., chairs, tables, hot drinks and snacks) with some engagement activities during the order collection days, such as cooking with gadgets, cooking with children, energy efficient cooking, saving time and money, composting. These activities encourage people to talk to each other, increasing social connections (e.g., talk, share vouchers, and come out together and shopping for each other) and promoting knowledge about food. |
| (Ep6) Community relationships & word of mouth | We reply on good community relationships to promote our project. A lot of people know us from their neighbours, friends, and family members (word of mouth). We recruit community researchers as part of our research team. They live and work within the community, and as gatekeepers, they have strong connections with local residents. They help us engage with community people and promote our research. |
| (Ep7) Convenient location | Our project is located at the heart of the community, within a 10-minute walking distance. It is intentionally separate from the community centre to avoid any stigma, as people often associate community centres with charity and individuals seeking help. |
| (Ep8) Choice to select the produce | At the market stall, people can choose the produce and the quantity they need. |
| (Ep9) Life routine | Our events (i.e., order & pickup days and monthly market) have become people’s life routine. People mark our dates in their calendar. |
|  |  |
| **Barriers** |  |
| (Bp1) Bad weather | Heavy rain or snow can reduce footfall. |
| (Bp2) Limited opening times | The order and collection days are only available once a fortnight from 10 AM to 12 PM, making it difficult for many working families to attend. |
| (Bp3) Cumbersome order & collection processes | Order and pick-up require two separate days, which can be challenging. |
| (Bp4) Short duration of the project | The short duration (12 months) negatively affects awareness of the project. People’s shopping behaviours will change after the project stops. Also, it makes data collection more difficult. |
| (Bp5) Low awareness of the project | This is especially true at the beginning of the project, due to its short duration and because people don’t read our information letters. |
| (Bp6) Limited monetary & human resources | Due to limited staff and resources, we currently open the market stall once a month and hold order and pickup days fortnightly. |
| (Bp7) Living alone | People who live alone don’t eat much, and they don’t want cook. Our FV bags are too much for them. Therefore, for these reasons they don’t attend to our project. |
| (Bp8) Low literacy & information overload | Many people don’t read the information letter because of language barriers or receiving too many letters everyday, which lead to low uptake rate. |
| (Bp9) Stigma | Some people feel it is unfair not to receive vouchers, while others feel offended by being given free vouchers since they earn enough and don’t want to be considered as disadvantaged. |
| (Bp10) Cost of living crisis | High energy costs and other expenses may influence people’s cooking habits. As a result, some individuals prefer not to cook for long periods or lack the resources to prepare and cook food properly. |
| (Bp11) Lacking time for cooking | Some people have multiple jobs and are looking after children; they simply don’t want to spend a long-time cooking. |
| (Bp12) Long-term illness & dietary restrictions | People suffer long term illness may not be able to come and have to reply on other people shopping for them. |
| (Bp13) Lack of professional resources | Professional resources such as sales training, marketing research to know the community demand, store improvement. We don’t have these resources to run market stall like professional business. Potentially, it negatively effects our uptake rate. |
| (Bp14) FV preferences | For many people, fruit and veg is unattractive. It's unknown. It's hard to make people excited about it. That's a general thing. That’s one of the reasons why our uptake rate is low. |

| **Reading** | |
| --- | --- |
| **Enablers** | *Explanation* |
| (Er1) Fresh & long-lasting quality | The overall quality of the produce is good and lasts longer compared to the produce in other supermarkets. |
| (Er2) Direct financial support | The scheme provides direct financial support to selected households, allowing them to save the money for other bills or expenses. It also offers flexibility, allowing people to either save up or spend all the vouchers at once. |
| (Er3) Easy to use voucher | The voucher is easy for participants to use; the research team scans it to help people order produce. So community people have more time to interact with each other or research team. However, this creates a significant workload for the research team. |
| (Er4) Convenient location | The community centre is within walking distance for selected households. Also, people can also stay outside for the market and don’t have to go inside, helping them avoid any discomfort or stigma. |
| (Er5) Social connection and education | The market stall and engagement activities encourage people to talk to each other, increasing social connections (e.g., talk, share vouchers, and come out together and shopping for each other) and promoting knowledge about food. |
| (Er6) Community relationships & word of mouth | People often don’t read promotional materials initially, so we rely on word of mouth. The community centre and supplier’s strong community reputation builds trust, and our local researchers conduct door-to-door outreach to engage effectively with residents. |
| (Er7) Choice to select the produce | People can choose what they want and how much they need. |
| (Er8) Life routine | The stall opens regularly every Saturday, and people mark the dates on their calendars. It has become part of their routine. |
| (Er9) A good variety of FVs | Alongside staples, we offer exotic fruits and vegetables, such as dragon fruit, mooli, and plantains. |
|  |  |
| **Barriers** |  |
| (Br1) Bad weather | Heavy rain can reduce footfall. But the weather is relatively good in Reading this year. |
| (Br2) Limited opening times | The stall operates for only 2-3 hours every Saturday morning, which is also too early for some families with children. |
| (Br3) Short duration of the project | The short duration (12 months) negatively affects awareness of the project. People’s shopping behaviours will change after the project stops. Also, it makes data collection more difficult. |
| (Br4) Low awareness of the project | This is especially true at the beginning of the project, due to its short duration and because people don’t read the letters. |
| (Br5) Produce selection process | People don’t know the price of what they are purchasing before weighing it, which can lead to discomfort, embarrassment, long queues and thus reduced visibility for the market. |
| (Br6) Limited monetary and human resources | The community centre is running the stall, which increases operating costs and workload. Staff and volunteers work during the week and also on Saturdays, leading many to feel burnt out. |
| (Br7) Low literacy and information overload | Many people don’t read the information letter because of language barriers or receiving too many letters everyday. |
| (Br8) Stigma | Some people feel it is unfair not to receive vouchers, while others feel offended by being given free vouchers since they earn enough and don’t want to be considered as disadvantaged. |
| (Br9) Cost of living crisis | High energy cost and other cost may change people’s cooking habit. |
| (Br10) Lacking time for cooking | Some people have multiple jobs and are looking after children; they simply don’t want to spend a long-time cooking. |
| (Br11) Long-term illness and dietary restrictions | People suffer long term illness may not be able to come and have to reply on other people shopping for them. |
| (Br12) High prices (for non-intervention group) | For the non-intervention group, the overall prices are higher than those at other supermarkets. Not many people buy the produce with cash. |
