# Supplementary material for: Modelling enablers and barriers to improve uptake of a fruit and vegetable voucher scheme (the fresh street community) in England: a TISM-MICMAC approach
Source: BMC Public Health. 2026 Mar 21;26:1399. doi: 10.1186/s12889-026-27063-3 (PMC13126962; doi:10.1186/s12889-026-27063-3)
Supplement: Supplementary file 2 — Supplementary Material 2. [file 12889_2026_27063_MOESM2_ESM.docx]

**Questionnaire for Plymouth**

| **Questions** | **Yes/No** | **In what way? Give reason in brief.** |
| --- | --- | --- |
| Fresh & long-lasting quality influence direct financial support |  |  |
| 。。。 influence easy to use voucher |  |  |
| 。。。 influence local and seasonal produce |  |  |
| 。。。 influence social connection and education |  |  |
| 。。。 influence community relationships & word of mouth |  |  |
| 。。。 influence convenient location |  |  |
| 。。。 influence choice to select the produce |  |  |
| 。。。 influence life routine |  |  |
| 。。。 influence bad weather |  |  |
| 。。。 influence limited opening times |  |  |
| 。。。 influence cumbersome order and collection processes |  |  |
| 。。。 influence short duration of the project |  |  |
| 。。。 influence low awareness of the project |  |  |
| 。。。 influence limited monetary and human resources |  |  |
| 。。。 influence living alone |  |  |
| 。。。 influence low literacy and information overload |  |  |
| 。。。 influence stigma |  |  |
| 。。。 influence cost of living crisis |  |  |
| 。。。 influence lacking time for cooking |  |  |
| 。。。 influence long-term illness and dietary restrictions |  |  |
| 。。。 influence lack of professional resources |  |  |
| 。。。 influence FV preferences |  |  |
|  |  |  |
| Direct financial support influence fresh & long-lasting quality |  |  |
| 。。。 influence easy to use voucher |  |  |
| 。。。 influence local and seasonal produce |  |  |
| 。。。 influence social connection and education |  |  |
| 。。。 influence community relationships & word of mouth |  |  |
| 。。。 influence convenient location |  |  |
| 。。。 influence choice to select the produce |  |  |
| 。。。 influence life routine |  |  |
| 。。。 influence bad weather |  |  |
| 。。。 influence limited opening times |  |  |
| 。。。 influence cumbersome order and collection processes |  |  |
| 。。。 influence short duration of the project |  |  |
| 。。。 influence low awareness of the project |  |  |
| 。。。 influence limited monetary and human resources |  |  |
| 。。。 influence living alone |  |  |
| 。。。 influence low literacy and information overload |  |  |
| 。。。 influence stigma |  |  |
| 。。。 influence cost of living crisis |  |  |
| 。。。 influence lacking time for cooking |  |  |
| 。。。 influence long-term illness and dietary restrictions |  |  |
| 。。。 influence lack of professional resources |  |  |
| 。。。 influence FV preferences |  |  |
|  |  |  |
| Easy to use voucher influence fresh & long-lasting quality |  |  |
| 。。。 influence direct financial support |  |  |
| 。。。 influence local and seasonal produce |  |  |
| 。。。 influence social connection and education |  |  |
| 。。。 influence community relationships & word of mouth |  |  |
| 。。。 influence convenient location |  |  |
| 。。。 influence choice to select the produce |  |  |
| 。。。 influence life routine |  |  |
| 。。。 influence bad weather |  |  |
| 。。。 influence limited opening times |  |  |
| 。。。 influence cumbersome order and collection processes |  |  |
| 。。。 influence short duration of the project |  |  |
| 。。。 influence low awareness of the project |  |  |
| 。。。 influence limited monetary and human resources |  |  |
| 。。。 influence living alone |  |  |
| 。。。 influence low literacy and information overload |  |  |
| 。。。 influence stigma |  |  |
| 。。。 influence cost of living crisis |  |  |
| 。。。 influence lacking time for cooking |  |  |
| 。。。 influence long-term illness and dietary restrictions |  |  |
| 。。。 influence lack of professional resources |  |  |
| 。。。 influence FV preferences |  |  |
|  |  |  |
| Local & seasonal produce influence fresh & long-lasting quality |  |  |
| 。。。 influence direct financial support |  |  |
| 。。。 influence easy to use voucher |  |  |
| 。。。 influence social connection and education |  |  |
| 。。。 influence community relationships & word of mouth |  |  |
| 。。。 influence convenient location |  |  |
| 。。。 influence choice to select the produce |  |  |
| 。。。 influence life routine |  |  |
| 。。。 influence bad weather |  |  |
| 。。。 influence limited opening times |  |  |
| 。。。 influence cumbersome order and collection processes |  |  |
| 。。。 influence short duration of the project |  |  |
| 。。。 influence low awareness of the project |  |  |
| 。。。 influence limited monetary and human resources |  |  |
| 。。。 influence living alone |  |  |
| 。。。 influence low literacy and information overload |  |  |
| 。。。 influence stigma |  |  |
| 。。。 influence cost of living crisis |  |  |
| 。。。 influence lacking time for cooking |  |  |
| 。。。 influence long-term illness and dietary restrictions |  |  |
| 。。。 influence lack of professional resources |  |  |
| 。。。 influence FV preferences |  |  |
|  |  |  |
| Social connection & education influence fresh & long-lasting quality |  |  |
| 。。。 influence direct financial support |  |  |
| 。。。 influence easy to use voucher |  |  |
| 。。。 influence local & seasonal produce |  |  |
| 。。。 influence community relationships & word of mouth |  |  |
| 。。。 influence convenient location |  |  |
| 。。。 influence choice to select the produce |  |  |
| 。。。 influence life routine |  |  |
| 。。。 influence bad weather |  |  |
| 。。。 influence limited opening times |  |  |
| 。。。 influence cumbersome order and collection processes |  |  |
| 。。。 influence short duration of the project |  |  |
| 。。。 influence low awareness of the project |  |  |
| 。。。 influence limited monetary and human resources |  |  |
| 。。。 influence living alone |  |  |
| 。。。 influence low literacy and information overload |  |  |
| 。。。 influence stigma |  |  |
| 。。。 influence cost of living crisis |  |  |
| 。。。 influence lacking time for cooking |  |  |
| 。。。 influence long-term illness and dietary restrictions |  |  |
| 。。。 influence lack of professional resources |  |  |
| 。。。 influence FV preferences |  |  |
|  |  |  |
| Community relationships & word of mouth influence fresh & long-lasting quality |  |  |
| 。。。 influence direct financial support |  |  |
| 。。。 influence easy to use voucher |  |  |
| 。。。 influence local & seasonal produce |  |  |
| 。。。 influence social connection & education |  |  |
| 。。。 influence convenient location |  |  |
| 。。。 influence choice to select the produce |  |  |
| 。。。 influence life routine |  |  |
| 。。。 influence bad weather |  |  |
| 。。。 influence limited opening times |  |  |
| 。。。 influence cumbersome order and collection processes |  |  |
| 。。。 influence short duration of the project |  |  |
| 。。。 influence low awareness of the project |  |  |
| 。。。 influence limited monetary and human resources |  |  |
| 。。。 influence living alone |  |  |
| 。。。 influence low literacy and information overload |  |  |
| 。。。 influence stigma |  |  |
| 。。。 influence cost of living crisis |  |  |
| 。。。 influence lacking time for cooking |  |  |
| 。。。 influence long-term illness and dietary restrictions |  |  |
| 。。。 influence lack of professional resources |  |  |
| 。。。 influence FV preferences |  |  |
|  |  |  |
| Convenient location influence fresh & long-lasting quality |  |  |
| 。。。 influence direct financial support |  |  |
| 。。。 influence easy to use voucher |  |  |
| 。。。 influence local & seasonal produce |  |  |
| 。。。 influence social connection & education |  |  |
| 。。。 influence community relationships & word of mouth |  |  |
| 。。。 influence choice to select the produce |  |  |
| 。。。 influence life routine |  |  |
| 。。。 influence bad weather |  |  |
| 。。。 influence limited opening times |  |  |
| 。。。 influence cumbersome order and collection processes |  |  |
| 。。。 influence short duration of the project |  |  |
| 。。。 influence low awareness of the project |  |  |
| 。。。 influence limited monetary and human resources |  |  |
| 。。。 influence living alone |  |  |
| 。。。 influence low literacy and information overload |  |  |
| 。。。 influence stigma |  |  |
| 。。。 influence cost of living crisis |  |  |
| 。。。 influence lacking time for cooking |  |  |
| 。。。 influence long-term illness and dietary restrictions |  |  |
| 。。。 influence lack of professional resources |  |  |
| 。。。 influence FV preferences |  |  |
|  |  |  |
| Choice to select the produce influence fresh & long-lasting quality |  |  |
| 。。。 influence direct financial support |  |  |
| 。。。 influence easy to use voucher |  |  |
| 。。。 influence local & seasonal produce |  |  |
| 。。。 influence social connection & education |  |  |
| 。。。 influence community relationships & word of mouth |  |  |
| 。。。 influence convenient location |  |  |
| 。。。 influence life routine |  |  |
| 。。。 influence bad weather |  |  |
| 。。。 influence limited opening times |  |  |
| 。。。 influence cumbersome order and collection processes |  |  |
| 。。。 influence short duration of the project |  |  |
| 。。。 influence low awareness of the project |  |  |
| 。。。 influence limited monetary and human resources |  |  |
| 。。。 influence living alone |  |  |
| 。。。 influence low literacy and information overload |  |  |
| 。。。 influence stigma |  |  |
| 。。。 influence cost of living crisis |  |  |
| 。。。 influence lacking time for cooking |  |  |
| 。。。 influence long-term illness and dietary restrictions |  |  |
| 。。。 influence lack of professional resources |  |  |
| 。。。 influence FV preferences |  |  |
|  |  |  |
| Life routine influence fresh & long-lasting quality |  |  |
| 。。。 influence direct financial support |  |  |
| 。。。 influence easy to use voucher |  |  |
| 。。。 influence local & seasonal produce |  |  |
| 。。。 influence social connection & education |  |  |
| 。。。 influence community relationships & word of mouth |  |  |
| 。。。 influence convenient location |  |  |
| 。。。 influence choice to select the produce |  |  |
| 。。。 influence bad weather |  |  |
| 。。。 influence limited opening times |  |  |
| 。。。 influence cumbersome order and collection processes |  |  |
| 。。。 influence short duration of the project |  |  |
| 。。。 influence low awareness of the project |  |  |
| 。。。 influence limited monetary and human resources |  |  |
| 。。。 influence living alone |  |  |
| 。。。 influence low literacy and information overload |  |  |
| 。。。 influence stigma |  |  |
| 。。。 influence cost of living crisis |  |  |
| 。。。 influence lacking time for cooking |  |  |
| 。。。 influence long-term illness and dietary restrictions |  |  |
| 。。。 influence lack of professional resources |  |  |
| 。。。 influence FV preferences |  |  |
|  |  |  |
| Bad weather influence fresh & long-lasting quality |  |  |
| 。。。 influence direct financial support |  |  |
| 。。。 influence easy to use voucher |  |  |
| 。。。 influence local & seasonal produce |  |  |
| 。。。 influence social connection & education |  |  |
| 。。。 influence community relationships & word of mouth |  |  |
| 。。。 influence convenient location |  |  |
| 。。。 influence choice to select the produce |  |  |
| 。。。 influence life routine |  |  |
| 。。。 influence limited opening times |  |  |
| 。。。 influence cumbersome order and collection processes |  |  |
| 。。。 influence short duration of the project |  |  |
| 。。。 influence low awareness of the project |  |  |
| 。。。 influence limited monetary and human resources |  |  |
| 。。。 influence living alone |  |  |
| 。。。 influence low literacy and information overload |  |  |
| 。。。 influence stigma |  |  |
| 。。。 influence cost of living crisis |  |  |
| 。。。 influence lacking time for cooking |  |  |
| 。。。 influence long-term illness and dietary restrictions |  |  |
| 。。。 influence lack of professional resources |  |  |
| 。。。 influence FV preferences |  |  |
|  |  |  |
| Limited opening times influence fresh & long-lasting quality |  |  |
| 。。。 influence direct financial support |  |  |
| 。。。 influence easy to use voucher |  |  |
| 。。。 influence local & seasonal produce |  |  |
| 。。。 influence social connection & education |  |  |
| 。。。 influence community relationships & word of mouth |  |  |
| 。。。 influence convenient location |  |  |
| 。。。 influence choice to select the produce |  |  |
| 。。。 influence life routine |  |  |
| 。。。 influence bad weather |  |  |
| 。。。 influence cumbersome order and collection processes |  |  |
| 。。。 influence short duration of the project |  |  |
| 。。。 influence low awareness of the project |  |  |
| 。。。 influence limited monetary and human resources |  |  |
| 。。。 influence living alone |  |  |
| 。。。 influence low literacy and information overload |  |  |
| 。。。 influence stigma |  |  |
| 。。。 influence cost of living crisis |  |  |
| 。。。 influence lacking time for cooking |  |  |
| 。。。 influence long-term illness and dietary restrictions |  |  |
| 。。。 influence lack of professional resources |  |  |
| 。。。 influence FV preferences |  |  |
|  |  |  |
| Cumbersome order and collection processes influence fresh & long-lasting quality |  |  |
| 。。。 influence direct financial support |  |  |
| 。。。 influence easy to use voucher |  |  |
| 。。。 influence local & seasonal produce |  |  |
| 。。。 influence social connection & education |  |  |
| 。。。 influence community relationships & word of mouth |  |  |
| 。。。 influence convenient location |  |  |
| 。。。 influence choice to select the produce |  |  |
| 。。。 influence life routine |  |  |
| 。。。 influence bad weather |  |  |
| 。。。 influence limited opening times |  |  |
| 。。。 influence short duration of the project |  |  |
| 。。。 influence low awareness of the project |  |  |
| 。。。 influence limited monetary and human resources |  |  |
| 。。。 influence living alone |  |  |
| 。。。 influence low literacy and information overload |  |  |
| 。。。 influence stigma |  |  |
| 。。。 influence cost of living crisis |  |  |
| 。。。 influence lacking time for cooking |  |  |
| 。。。 influence long-term illness and dietary restrictions |  |  |
| 。。。 influence lack of professional resources |  |  |
| 。。。 influence FV preferences |  |  |
|  |  |  |
| Short duration of the project influence fresh & long-lasting quality |  |  |
| 。。。 influence direct financial support |  |  |
| 。。。 influence easy to use voucher |  |  |
| 。。。 influence local & seasonal produce |  |  |
| 。。。 influence social connection & education |  |  |
| 。。。 influence community relationships & word of mouth |  |  |
| 。。。 influence convenient location |  |  |
| 。。。 influence choice to select the produce |  |  |
| 。。。 influence life routine |  |  |
| 。。。 influence bad weather |  |  |
| 。。。 influence limited opening times |  |  |
| 。。。 influence cumbersome order and collection processes |  |  |
| 。。。 influence low awareness of the project |  |  |
| 。。。 influence limited monetary and human resources |  |  |
| 。。。 influence living alone |  |  |
| 。。。 influence low literacy and information overload |  |  |
| 。。。 influence stigma |  |  |
| 。。。 influence cost of living crisis |  |  |
| 。。。 influence lacking time for cooking |  |  |
| 。。。 influence long-term illness and dietary restrictions |  |  |
| 。。。 influence lack of professional resources |  |  |
| 。。。 influence FV preferences |  |  |
|  |  |  |
| Low awareness of the project influence fresh & long-lasting quality |  |  |
| 。。。 influence direct financial support |  |  |
| 。。。 influence easy to use voucher |  |  |
| 。。。 influence local & seasonal produce |  |  |
| 。。。 influence social connection & education |  |  |
| 。。。 influence community relationships & word of mouth |  |  |
| 。。。 influence convenient location |  |  |
| 。。。 influence choice to select the produce |  |  |
| 。。。 influence life routine |  |  |
| 。。。 influence bad weather |  |  |
| 。。。 influence limited opening times |  |  |
| 。。。 influence cumbersome order and collection processes |  |  |
| 。。。 influence short duration of the project |  |  |
| 。。。 influence limited monetary and human resources |  |  |
| 。。。 influence living alone |  |  |
| 。。。 influence low literacy and information overload |  |  |
| 。。。 influence stigma |  |  |
| 。。。 influence cost of living crisis |  |  |
| 。。。 influence lacking time for cooking |  |  |
| 。。。 influence long-term illness and dietary restrictions |  |  |
| 。。。 influence lack of professional resources |  |  |
| 。。。 influence FV preferences |  |  |
|  |  |  |
| Limited monetary & human resources influence fresh & long-lasting quality |  |  |
| 。。。 influence direct financial support |  |  |
| 。。。 influence easy to use voucher |  |  |
| 。。。 influence local & seasonal produce |  |  |
| 。。。 influence social connection & education |  |  |
| 。。。 influence community relationships & word of mouth |  |  |
| 。。。 influence convenient location |  |  |
| 。。。 influence choice to select the produce |  |  |
| 。。。 influence life routine |  |  |
| 。。。 influence bad weather |  |  |
| 。。。 influence limited opening times |  |  |
| 。。。 influence cumbersome order and collection processes |  |  |
| 。。。 influence short duration of the project |  |  |
| 。。。 influence low awareness of the project |  |  |
| 。。。 influence living alone |  |  |
| 。。。 influence low literacy and information overload |  |  |
| 。。。 influence stigma |  |  |
| 。。。 influence cost of living crisis |  |  |
| 。。。 influence lacking time for cooking |  |  |
| 。。。 influence long-term illness and dietary restrictions |  |  |
| 。。。 influence lack of professional resources |  |  |
| 。。。 influence FV preferences |  |  |
|  |  |  |
| Living alone influence fresh & long-lasting quality |  |  |
| 。。。 influence direct financial support |  |  |
| 。。。 influence easy to use voucher |  |  |
| 。。。 influence local & seasonal produce |  |  |
| 。。。 influence social connection & education |  |  |
| 。。。 influence community relationships & word of mouth |  |  |
| 。。。 influence convenient location |  |  |
| 。。。 influence choice to select the produce |  |  |
| 。。。 influence life routine |  |  |
| 。。。 influence bad weather |  |  |
| 。。。 influence limited opening times |  |  |
| 。。。 influence cumbersome order and collection processes |  |  |
| 。。。 influence short duration of the project |  |  |
| 。。。 influence low awareness of the project |  |  |
| 。。。 influence limited monetary & human resources |  |  |
| 。。。 influence low literacy and information overload |  |  |
| 。。。 influence stigma |  |  |
| 。。。 influence cost of living crisis |  |  |
| 。。。 influence lacking time for cooking |  |  |
| 。。。 influence long-term illness and dietary restrictions |  |  |
| 。。。 influence lack of professional resources |  |  |
| 。。。 influence FV preferences |  |  |
|  |  |  |
| Low literacy and information overload influence fresh & long-lasting quality |  |  |
| 。。。 influence direct financial support |  |  |
| 。。。 influence easy to use voucher |  |  |
| 。。。 influence local & seasonal produce |  |  |
| 。。。 influence social connection & education |  |  |
| 。。。 influence community relationships & word of mouth |  |  |
| 。。。 influence convenient location |  |  |
| 。。。 influence choice to select the produce |  |  |
| 。。。 influence life routine |  |  |
| 。。。 influence bad weather |  |  |
| 。。。 influence limited opening times |  |  |
| 。。。 influence cumbersome order and collection processes |  |  |
| 。。。 influence short duration of the project |  |  |
| 。。。 influence low awareness of the project |  |  |
| 。。。 influence limited monetary & human resources |  |  |
| 。。。 influence living alone |  |  |
| 。。。 influence stigma |  |  |
| 。。。 influence cost of living crisis |  |  |
| 。。。 influence lacking time for cooking |  |  |
| 。。。 influence long-term illness and dietary restrictions |  |  |
| 。。。 influence lack of professional resources |  |  |
| 。。。 influence FV preferences |  |  |
|  |  |  |
| Stigma influence fresh & long-lasting quality |  |  |
| 。。。 influence direct financial support |  |  |
| 。。。 influence easy to use voucher |  |  |
| 。。。 influence local & seasonal produce |  |  |
| 。。。 influence social connection & education |  |  |
| 。。。 influence community relationships & word of mouth |  |  |
| 。。。 influence convenient location |  |  |
| 。。。 influence choice to select the produce |  |  |
| 。。。 influence life routine |  |  |
| 。。。 influence bad weather |  |  |
| 。。。 influence limited opening times |  |  |
| 。。。 influence cumbersome order and collection processes |  |  |
| 。。。 influence short duration of the project |  |  |
| 。。。 influence low awareness of the project |  |  |
| 。。。 influence limited monetary & human resources |  |  |
| 。。。 influence living alone |  |  |
| 。。。 influence low literacy and information overload |  |  |
| 。。。 influence cost of living crisis |  |  |
| 。。。 influence lacking time for cooking |  |  |
| 。。。 influence long-term illness and dietary restrictions |  |  |
| 。。。 influence lack of professional resources |  |  |
| 。。。 influence FV preferences |  |  |
|  |  |  |
| Cost of living crisis influence fresh & long-lasting quality |  |  |
| 。。。 influence direct financial support |  |  |
| 。。。 influence easy to use voucher |  |  |
| 。。。 influence local & seasonal produce |  |  |
| 。。。 influence social connection & education |  |  |
| 。。。 influence community relationships & word of mouth |  |  |
| 。。。 influence convenient location |  |  |
| 。。。 influence choice to select the produce |  |  |
| 。。。 influence life routine |  |  |
| 。。。 influence bad weather |  |  |
| 。。。 influence limited opening times |  |  |
| 。。。 influence cumbersome order and collection processes |  |  |
| 。。。 influence short duration of the project |  |  |
| 。。。 influence low awareness of the project |  |  |
| 。。。 influence limited monetary & human resources |  |  |
| 。。。 influence living alone |  |  |
| 。。。 influence low literacy and information overload |  |  |
| 。。。 influence stigma |  |  |
| 。。。 influence lacking time for cooking |  |  |
| 。。。 influence long-term illness and dietary restrictions |  |  |
| 。。。 influence lack of professional resources |  |  |
| 。。。 influence FV preferences |  |  |
|  |  |  |
| Lacking time for cooking influence fresh & long-lasting quality |  |  |
| 。。。 influence direct financial support |  |  |
| 。。。 influence easy to use voucher |  |  |
| 。。。 influence local & seasonal produce |  |  |
| 。。。 influence social connection & education |  |  |
| 。。。 influence community relationships & word of mouth |  |  |
| 。。。 influence convenient location |  |  |
| 。。。 influence choice to select the produce |  |  |
| 。。。 influence life routine |  |  |
| 。。。 influence bad weather |  |  |
| 。。。 influence limited opening times |  |  |
| 。。。 influence cumbersome order and collection processes |  |  |
| 。。。 influence short duration of the project |  |  |
| 。。。 influence low awareness of the project |  |  |
| 。。。 influence limited monetary & human resources |  |  |
| 。。。 influence living alone |  |  |
| 。。。 influence low literacy and information overload |  |  |
| 。。。 influence stigma |  |  |
| 。。。 influence cost of living crisis |  |  |
| 。。。 influence long-term illness and dietary restrictions |  |  |
| 。。。 influence lack of professional resources |  |  |
| 。。。 influence FV preferences |  |  |
|  |  |  |
| Long-term illness and dietary restrictions influence fresh & long-lasting quality |  |  |
| 。。。 influence direct financial support |  |  |
| 。。。 influence easy to use voucher |  |  |
| 。。。 influence local & seasonal produce |  |  |
| 。。。 influence social connection & education |  |  |
| 。。。 influence community relationships & word of mouth |  |  |
| 。。。 influence convenient location |  |  |
| 。。。 influence choice to select the produce |  |  |
| 。。。 influence life routine |  |  |
| 。。。 influence bad weather |  |  |
| 。。。 influence limited opening times |  |  |
| 。。。 influence cumbersome order and collection processes |  |  |
| 。。。 influence short duration of the project |  |  |
| 。。。 influence low awareness of the project |  |  |
| 。。。 influence limited monetary & human resources |  |  |
| 。。。 influence living alone |  |  |
| 。。。 influence low literacy and information overload |  |  |
| 。。。 influence stigma |  |  |
| 。。。 influence cost of living crisis |  |  |
| 。。。 influence lacking time for cooking |  |  |
| 。。。 influence lack of professional resources |  |  |
| 。。。 influence FV preferences |  |  |
|  |  |  |
| Lack of professional resources influence fresh & long-lasting quality |  |  |
| 。。。 influence direct financial support |  |  |
| 。。。 influence easy to use voucher |  |  |
| 。。。 influence local & seasonal produce |  |  |
| 。。。 influence social connection & education |  |  |
| 。。。 influence community relationships & word of mouth |  |  |
| 。。。 influence convenient location |  |  |
| 。。。 influence choice to select the produce |  |  |
| 。。。 influence life routine |  |  |
| 。。。 influence bad weather |  |  |
| 。。。 influence limited opening times |  |  |
| 。。。 influence cumbersome order and collection processes |  |  |
| 。。。 influence short duration of the project |  |  |
| 。。。 influence low awareness of the project |  |  |
| 。。。 influence limited monetary & human resources |  |  |
| 。。。 influence living alone |  |  |
| 。。。 influence low literacy and information overload |  |  |
| 。。。 influence stigma |  |  |
| 。。。 influence cost of living crisis |  |  |
| 。。。 influence lacking time for cooking |  |  |
| 。。。 influence long-term illness and dietary restrictions |  |  |
| 。。。 influence FV preferences |  |  |
|  |  |  |
| FV preferences influence fresh & long-lasting quality |  |  |
| 。。。 influence direct financial support |  |  |
| 。。。 influence easy to use voucher |  |  |
| 。。。 influence local & seasonal produce |  |  |
| 。。。 influence social connection & education |  |  |
| 。。。 influence community relationships & word of mouth |  |  |
| 。。。 influence convenient location |  |  |
| 。。。 influence choice to select the produce |  |  |
| 。。。 influence life routine |  |  |
| 。。。 influence bad weather |  |  |
| 。。。 influence limited opening times |  |  |
| 。。。 influence cumbersome order and collection processes |  |  |
| 。。。 influence short duration of the project |  |  |
| 。。。 influence low awareness of the project |  |  |
| 。。。 influence limited monetary & human resources |  |  |
| 。。。 influence living alone |  |  |
| 。。。 influence low literacy and information overload |  |  |
| 。。。 influence stigma |  |  |
| 。。。 influence cost of living crisis |  |  |
| 。。。 influence lacking time for cooking |  |  |
| 。。。 influence long-term illness and dietary restrictions |  |  |
| 。。。 influence lack of professional resources |  |  |
|  |  |  |

**Questionnaire for Reading**

| **Questions** | **Yes/No** | **In what way? Give reason in brief.** |
| --- | --- | --- |
| Fresh & long-lasting quality influences direct financial support |  |  |
| 。。。 influence easy to use voucher |  |  |
| 。。。 influence social connection and education |  |  |
| 。。。 influence community relationships & word of mouth |  |  |
| 。。。 influence convenient location |  |  |
| 。。。 influence choice to select the produce |  |  |
| 。。。 influence a good variety of FVs |  |  |
| 。。。 influence life routine |  |  |
| 。。。 influence bad weather |  |  |
| 。。。 influence limited opening times |  |  |
| 。。。 influence produce selection process |  |  |
| 。。。 influence short duration of the project |  |  |
| 。。。 influence low awareness of the project |  |  |
| 。。。 influence limited monetary and human resources |  |  |
| 。。。 influence low literacy and information overload |  |  |
| 。。。 influence stigma |  |  |
| 。。。 influence cost of living crisis |  |  |
| 。。。 influence lacking time for cooking |  |  |
| 。。。 influence high prices (for non-intervention group) |  |  |
| 。。。 influence long-term illness and dietary restrictions |  |  |
|  |  |  |
| Direct financial support influences fresh & long-lasting quality |  |  |
| 。。。 influences easy to use voucher |  |  |
| 。。。 influences social connection and education |  |  |
| 。。。 influences community relationships & word of mouth |  |  |
| 。。。 influences convenient location |  |  |
| 。。。 influences choice to select the produce |  |  |
| 。。。 influences a good variety of FVs |  |  |
| 。。。 influences life routine |  |  |
| 。。。 influences bad weather |  |  |
| 。。。 influences limited opening times |  |  |
| 。。。 influences produce selection process |  |  |
| 。。。 influences short duration of the project |  |  |
| 。。。 influences low awareness of the project |  |  |
| 。。。 influences limited monetary and human resources |  |  |
| 。。。 influences low literacy and information overload |  |  |
| 。。。 influences stigma |  |  |
| 。。。 influences cost of living crisis |  |  |
| 。。。 influences lacking time for cooking |  |  |
| 。。。 influences high prices (for non-intervention group) |  |  |
| 。。。 influences long-term illness and dietary restrictions |  |  |
|  |  |  |
| Easy to use voucher influences fresh & long-lasting quality |  |  |
| 。。。 influences direct financial support |  |  |
| 。。。 influences social connection and education |  |  |
| 。。。 influences community relationships & word of mouth |  |  |
| 。。。 influences convenient location |  |  |
| 。。。 influences choice to select the produce |  |  |
| 。。。 influences a good variety of FVs |  |  |
| 。。。 influences life routine |  |  |
| 。。。 influences bad weather |  |  |
| 。。。 influences limited opening times |  |  |
| 。。。 influences produce selection process |  |  |
| 。。。 influences short duration of the project |  |  |
| 。。。 influences low awareness of the project |  |  |
| 。。。 influences limited monetary and human resources |  |  |
| 。。。 influences low literacy and information overload |  |  |
| 。。。 influences stigma |  |  |
| 。。。 influences cost of living crisis |  |  |
| 。。。 influences lacking time for cooking |  |  |
| 。。。 influences high prices (for non-intervention group) |  |  |
| 。。。 influences long-term illness and dietary restrictions |  |  |
|  |  |  |
| Social connection & education influence fresh & long-lasting quality |  |  |
| 。。。 influence direct financial support |  |  |
| 。。。 influence easy to use voucher |  |  |
| 。。。 influence community relationships & word of mouth |  |  |
| 。。。 influence convenient location |  |  |
| 。。。 influence choice to select the produce |  |  |
| 。。。 influence a good variety of FVs |  |  |
| 。。。 influence life routine |  |  |
| 。。。 influence bad weather |  |  |
| 。。。 influence limited opening times |  |  |
| 。。。 influence produce selection process |  |  |
| 。。。 influence short duration of the project |  |  |
| 。。。 influence low awareness of the project |  |  |
| 。。。 influence limited monetary and human resources |  |  |
| 。。。 influence low literacy and information overload |  |  |
| 。。。 influence stigma |  |  |
| 。。。 influence cost of living crisis |  |  |
| 。。。 influence lacking time for cooking |  |  |
| 。。。 influence high prices (for non-intervention group) |  |  |
| 。。。 influence long-term illness and dietary restrictions |  |  |
|  |  |  |
| Community relationships & word of mouth influence fresh & long-lasting quality |  |  |
| 。。。 influence direct financial support |  |  |
| 。。。 influence easy to use voucher |  |  |
| 。。。 influence social connection & education |  |  |
| 。。。 influence convenient location |  |  |
| 。。。 influence choice to select the produce |  |  |
| 。。。 influence a good variety of FVs |  |  |
| 。。。 influence life routine |  |  |
| 。。。 influence bad weather |  |  |
| 。。。 influence limited opening times |  |  |
| 。。。 influence produce selection process |  |  |
| 。。。 influence short duration of the project |  |  |
| 。。。 influence low awareness of the project |  |  |
| 。。。 influence limited monetary and human resources |  |  |
| 。。。 influence low literacy and information overload |  |  |
| 。。。 influence stigma |  |  |
| 。。。 influence cost of living crisis |  |  |
| 。。。 influence lacking time for cooking |  |  |
| 。。。 influence high prices (for non-intervention group) |  |  |
| 。。。 influence long-term illness and dietary restrictions |  |  |
|  |  |  |
| Convenient location influences fresh & long-lasting quality |  |  |
| 。。。 influences direct financial support |  |  |
| 。。。 influences easy to use voucher |  |  |
| 。。。 influences social connection & education |  |  |
| 。。。 influences community relationships & word of mouth |  |  |
| 。。。 influences choice to select the produce |  |  |
| 。。。 influences a good variety of FVs |  |  |
| 。。。 influences life routine |  |  |
| 。。。 influences bad weather |  |  |
| 。。。 influences limited opening times |  |  |
| 。。。 influences produce selection process |  |  |
| 。。。 influences short duration of the project |  |  |
| 。。。 influences low awareness of the project |  |  |
| 。。。 influences limited monetary and human resources |  |  |
| 。。。 influences low literacy and information overload |  |  |
| 。。。 influences stigma |  |  |
| 。。。 influences cost of living crisis |  |  |
| 。。。 influences lacking time for cooking |  |  |
| 。。。 influences high prices (for non-intervention group) |  |  |
| 。。。 influences long-term illness and dietary restrictions |  |  |
|  |  |  |
| Choice to select the produce influences fresh & long-lasting quality |  |  |
| 。。。 influences direct financial support |  |  |
| 。。。 influences easy to use voucher |  |  |
| 。。。 influences social connection & education |  |  |
| 。。。 influences community relationships & word of mouth |  |  |
| 。。。 influences convenient location |  |  |
| 。。。 influences a good variety of FVs |  |  |
| 。。。 influences life routine |  |  |
| 。。。 influences bad weather |  |  |
| 。。。 influences limited opening times |  |  |
| 。。。 influences produce selection process |  |  |
| 。。。 influences short duration of the project |  |  |
| 。。。 influences low awareness of the project |  |  |
| 。。。 influences limited monetary and human resources |  |  |
| 。。。 influences low literacy and information overload |  |  |
| 。。。 influences stigma |  |  |
| 。。。 influences cost of living crisis |  |  |
| 。。。 influences lacking time for cooking |  |  |
| 。。。 influences high prices (for non-intervention group) |  |  |
| 。。。 influences long-term illness and dietary restrictions |  |  |
|  |  |  |
| A good variety of FVs influences fresh & long-lasting quality |  |  |
| 。。。 influences direct financial support |  |  |
| 。。。 influences easy to use voucher |  |  |
| 。。。 influences social connection & education |  |  |
| 。。。 influences community relationships & word of mouth |  |  |
| 。。。 influences convenient location |  |  |
| 。。。 influences choice to select the produce |  |  |
| 。。。 influences life routine |  |  |
| 。。。 influences bad weather |  |  |
| 。。。 influences limited opening times |  |  |
| 。。。 influences produce selection process |  |  |
| 。。。 influences short duration of the project |  |  |
| 。。。 influences low awareness of the project |  |  |
| 。。。 influences limited monetary and human resources |  |  |
| 。。。 influences low literacy and information overload |  |  |
| 。。。 influences stigma |  |  |
| 。。。 influences cost of living crisis |  |  |
| 。。。 influences lacking time for cooking |  |  |
| 。。。 influences high prices (for non-intervention group) |  |  |
| 。。。 influences long-term illness and dietary restrictions |  |  |
|  |  |  |
| Life routine influences fresh & long-lasting quality |  |  |
| 。。。 influences direct financial support |  |  |
| 。。。 influences easy to use voucher |  |  |
| 。。。 influences social connection & education |  |  |
| 。。。 influences community relationships & word of mouth |  |  |
| 。。。 influences convenient location |  |  |
| 。。。 influences choice to select the produce |  |  |
| 。。。 influences a good variety of FVs |  |  |
| 。。。 influences bad weather |  |  |
| 。。。 influences limited opening times |  |  |
| 。。。 influences produce selection process |  |  |
| 。。。 influences short duration of the project |  |  |
| 。。。 influences low awareness of the project |  |  |
| 。。。 influences limited monetary and human resources |  |  |
| 。。。 influences low literacy and information overload |  |  |
| 。。。 influences stigma |  |  |
| 。。。 influences cost of living crisis |  |  |
| 。。。 influences lacking time for cooking |  |  |
| 。。。 influences high prices (for non-intervention group) |  |  |
| 。。。 influences long-term illness and dietary restrictions |  |  |
|  |  |  |
| Bad weather influences fresh & long-lasting quality |  |  |
| 。。。 influences direct financial support |  |  |
| 。。。 influences easy to use voucher |  |  |
| 。。。 influences social connection & education |  |  |
| 。。。 influences community relationships & word of mouth |  |  |
| 。。。 influences convenient location |  |  |
| 。。。 influences choice to select the produce |  |  |
| 。。。 influences a good variety of FVs |  |  |
| 。。。 influences life routine |  |  |
| 。。。 influences limited opening times |  |  |
| 。。。 influences produce selection process |  |  |
| 。。。 influences short duration of the project |  |  |
| 。。。 influences low awareness of the project |  |  |
| 。。。 influences limited monetary and human resources |  |  |
| 。。。 influences low literacy and information overload |  |  |
| 。。。 influences stigma |  |  |
| 。。。 influences cost of living crisis |  |  |
| 。。。 influences lacking time for cooking |  |  |
| 。。。 influences high prices (for non-intervention group) |  |  |
| 。。。 influences long-term illness and dietary restrictions |  |  |
|  |  |  |
| Limited opening times influence fresh & long-lasting quality |  |  |
| 。。。 influence direct financial support |  |  |
| 。。。 influence easy to use voucher |  |  |
| 。。。 influence social connection & education |  |  |
| 。。。 influence community relationships & word of mouth |  |  |
| 。。。 influence convenient location |  |  |
| 。。。 influence choice to select the produce |  |  |
| 。。。 influence a good variety of FVs |  |  |
| 。。。 influence life routine |  |  |
| 。。。 influence bad weather |  |  |
| 。。。 influence produce selection process |  |  |
| 。。。 influence short duration of the project |  |  |
| 。。。 influence low awareness of the project |  |  |
| 。。。 influence limited monetary and human resources |  |  |
| 。。。 influence low literacy and information overload |  |  |
| 。。。 influence stigma |  |  |
| 。。。 influence cost of living crisis |  |  |
| 。。。 influence lacking time for cooking |  |  |
| 。。。 influence high prices (for non-intervention group) |  |  |
| 。。。 influence long-term illness and dietary restrictions |  |  |
|  |  |  |
| Produce selection process influence fresh & long-lasting quality |  |  |
| 。。。 influence direct financial support |  |  |
| 。。。 influence easy to use voucher |  |  |
| 。。。 influence social connection & education |  |  |
| 。。。 influence community relationships & word of mouth |  |  |
| 。。。 influence convenient location |  |  |
| 。。。 influence choice to select the produce |  |  |
| 。。。 influence a good variety of FVs |  |  |
| 。。。 influence life routine |  |  |
| 。。。 influence bad weather |  |  |
| 。。。 influence limited opening times |  |  |
| 。。。 influence short duration of the project |  |  |
| 。。。 influence low awareness of the project |  |  |
| 。。。 influence limited monetary and human resources |  |  |
| 。。。 influence low literacy and information overload |  |  |
| 。。。 influence stigma |  |  |
| 。。。 influence cost of living crisis |  |  |
| 。。。 influence lacking time for cooking |  |  |
| 。。。 influence high prices (for non-intervention group) |  |  |
| 。。。 influence long-term illness and dietary restrictions |  |  |
|  |  |  |
| Short duration of the project influences fresh & long-lasting quality |  |  |
| 。。。 influences direct financial support |  |  |
| 。。。 influences easy to use voucher |  |  |
| 。。。 influences social connection & education |  |  |
| 。。。 influences community relationships & word of mouth |  |  |
| 。。。 influences convenient location |  |  |
| 。。。 influences choice to select the produce |  |  |
| 。。。 influences a good variety of FVs |  |  |
| 。。。 influences life routine |  |  |
| 。。。 influences bad weather |  |  |
| 。。。 influences limited opening times |  |  |
| 。。。 influences produce selection process |  |  |
| 。。。 influences low awareness of the project |  |  |
| 。。。 influences limited monetary and human resources |  |  |
| 。。。 influences low literacy and information overload |  |  |
| 。。。 influences stigma |  |  |
| 。。。 influences cost of living crisis |  |  |
| 。。。 influences lacking time for cooking |  |  |
| 。。。 influences high prices (for non-intervention group) |  |  |
| 。。。 influences long-term illness and dietary restrictions |  |  |
|  |  |  |
| Low awareness of the project influences fresh & long-lasting quality |  |  |
| 。。。 influences direct financial support |  |  |
| 。。。 influences easy to use voucher |  |  |
| 。。。 influences social connection & education |  |  |
| 。。。 influences community relationships & word of mouth |  |  |
| 。。。 influences convenient location |  |  |
| 。。。 influences choice to select the produce |  |  |
| 。。。 influences a good variety of FVs |  |  |
| 。。。 influences life routine |  |  |
| 。。。 influences bad weather |  |  |
| 。。。 influences limited opening times |  |  |
| 。。。 influences produce selection process |  |  |
| 。。。 influences short duration of the project |  |  |
| 。。。 influences limited monetary and human resources |  |  |
| 。。。 influences low literacy and information overload |  |  |
| 。。。 influences stigma |  |  |
| 。。。 influences cost of living crisis |  |  |
| 。。。 influences lacking time for cooking |  |  |
| 。。。 influences high prices (for non-intervention group) |  |  |
| 。。。 influences long-term illness and dietary restrictions |  |  |
|  |  |  |
| Limited monetary & human resources influences fresh & long-lasting quality |  |  |
| 。。。 influences direct financial support |  |  |
| 。。。 influences easy to use voucher |  |  |
| 。。。 influences social connection & education |  |  |
| 。。。 influences community relationships & word of mouth |  |  |
| 。。。 influences convenient location |  |  |
| 。。。 influences choice to select the produce |  |  |
| 。。。 influences a good variety of FVs |  |  |
| 。。。 influences life routine |  |  |
| 。。。 influences bad weather |  |  |
| 。。。 influences limited opening times |  |  |
| 。。。 influences produce selection process |  |  |
| 。。。 influences short duration of the project |  |  |
| 。。。 influences low awareness of the project |  |  |
| 。。。 influences low literacy and information overload |  |  |
| 。。。 influences stigma |  |  |
| 。。。 influences cost of living crisis |  |  |
| 。。。 influences lacking time for cooking |  |  |
| 。。。 influences high prices (for non-intervention group) |  |  |
| 。。。 influences long-term illness and dietary restrictions |  |  |
|  |  |  |
| Low literacy and information overload influence fresh & long-lasting quality |  |  |
| 。。。 influence direct financial support |  |  |
| 。。。 influence easy to use voucher |  |  |
| 。。。 influence social connection & education |  |  |
| 。。。 influence community relationships & word of mouth |  |  |
| 。。。 influence convenient location |  |  |
| 。。。 influence choice to select the produce |  |  |
| 。。。 influence a good variety of FVs |  |  |
| 。。。 influence life routine |  |  |
| 。。。 influence bad weather |  |  |
| 。。。 influence limited opening times |  |  |
| 。。。 influence produce selection process |  |  |
| 。。。 influence short duration of the project |  |  |
| 。。。 influence low awareness of the project |  |  |
| 。。。 influence limited monetary & human resources |  |  |
| 。。。 influence stigma |  |  |
| 。。。 influence cost of living crisis |  |  |
| 。。。 influence lacking time for cooking |  |  |
| 。。。 influence high prices (for non-intervention group) |  |  |
| 。。。 influence long-term illness and dietary restrictions |  |  |
|  |  |  |
| Stigma influence fresh & long-lasting quality |  |  |
| 。。。 influence direct financial support |  |  |
| 。。。 influence easy to use voucher |  |  |
| 。。。 influence social connection & education |  |  |
| 。。。 influence community relationships & word of mouth |  |  |
| 。。。 influence convenient location |  |  |
| 。。。 influence choice to select the produce |  |  |
| 。。。 influence a good variety of FVs |  |  |
| 。。。 influence life routine |  |  |
| 。。。 influence bad weather |  |  |
| 。。。 influence limited opening times |  |  |
| 。。。 influence produce selection process |  |  |
| 。。。 influence short duration of the project |  |  |
| 。。。 influence low awareness of the project |  |  |
| 。。。 influence limited monetary & human resources |  |  |
| 。。。 influence low literacy and information overload |  |  |
| 。。。 influence cost of living crisis |  |  |
| 。。。 influence lacking time for cooking |  |  |
| 。。。 influence high prices (for non-intervention group) |  |  |
| 。。。 influence long-term illness and dietary restrictions |  |  |
|  |  |  |
| Cost of living crisis influences fresh & long-lasting quality |  |  |
| 。。。 influences direct financial support |  |  |
| 。。。 influences easy to use voucher |  |  |
| 。。。 influences social connection & education |  |  |
| 。。。 influences community relationships & word of mouth |  |  |
| 。。。 influences convenient location |  |  |
| 。。。 influences choice to select the produce |  |  |
| 。。。 influences a good variety of FVs |  |  |
| 。。。 influences life routine |  |  |
| 。。。 influences bad weather |  |  |
| 。。。 influences limited opening times |  |  |
| 。。。 influences produce selection process |  |  |
| 。。。 influences short duration of the project |  |  |
| 。。。 influences low awareness of the project |  |  |
| 。。。 influences limited monetary & human resources |  |  |
| 。。。 influences low literacy and information overload |  |  |
| 。。。 influences stigma |  |  |
| 。。。 influences lacking time for cooking |  |  |
| 。。。 influences high prices (for non-intervention group) |  |  |
| 。。。 influences long-term illness and dietary restrictions |  |  |
|  |  |  |
| Lacking time for cooking influences fresh & long-lasting quality |  |  |
| 。。。 influences direct financial support |  |  |
| 。。。 influences easy to use voucher |  |  |
| 。。。 influences social connection & education |  |  |
| 。。。 influences community relationships & word of mouth |  |  |
| 。。。 influences convenient location |  |  |
| 。。。 influences choice to select the produce |  |  |
| 。。。 influences a good variety of FVs |  |  |
| 。。。 influences life routine |  |  |
| 。。。 influences bad weather |  |  |
| 。。。 influences limited opening times |  |  |
| 。。。 influences produce selection process |  |  |
| 。。。 influences short duration of the project |  |  |
| 。。。 influences low awareness of the project |  |  |
| 。。。 influences limited monetary & human resources |  |  |
| 。。。 influences low literacy and information overload |  |  |
| 。。。 influences stigma |  |  |
| 。。。 influences cost of living crisis |  |  |
| 。。。 influences high prices (for non-intervention group) |  |  |
| 。。。 influences long-term illness and dietary restrictions |  |  |
|  |  |  |
| High prices (for non-intervention group) influences fresh & long-lasting quality |  |  |
| 。。。 influences direct financial support |  |  |
| 。。。 influences easy to use voucher |  |  |
| 。。。 influences social connection & education |  |  |
| 。。。 influences community relationships & word of mouth |  |  |
| 。。。 influences convenient location |  |  |
| 。。。 influences choice to select the produce |  |  |
| 。。。 influences a good variety of FVs |  |  |
| 。。。 influences life routine |  |  |
| 。。。 influences bad weather |  |  |
| 。。。 influences limited opening times |  |  |
| 。。。 influences produce selection process |  |  |
| 。。。 influences short duration of the project |  |  |
| 。。。 influences low awareness of the project |  |  |
| 。。。 influences limited monetary & human resources |  |  |
| 。。。 influences low literacy and information overload |  |  |
| 。。。 influences stigma |  |  |
| 。。。 influences cost of living crisis |  |  |
| 。。。 influences lacking time for cooking |  |  |
| 。。。 influences long-term illness and dietary restrictions |  |  |
|  |  |  |
| Long-term illness and dietary restrictions influence fresh & long-lasting quality |  |  |
| 。。。 influence direct financial support |  |  |
| 。。。 influence easy to use voucher |  |  |
| 。。。 influence social connection & education |  |  |
| 。。。 influence community relationships & word of mouth |  |  |
| 。。。 influence convenient location |  |  |
| 。。。 influence choice to select the produce |  |  |
| 。。。 influence a good variety of FVs |  |  |
| 。。。 influence life routine |  |  |
| 。。。 influence bad weather |  |  |
| 。。。 influence limited opening times |  |  |
| 。。。 influence produce selection process |  |  |
| 。。。 influence short duration of the project |  |  |
| 。。。 influence low awareness of the project |  |  |
| 。。。 influence limited monetary & human resources |  |  |
| 。。。 influence low literacy and information overload |  |  |
| 。。。 influence stigma |  |  |
| 。。。 influence cost of living crisis |  |  |
| 。。。 influence lacking time for cooking |  |  |
| 。。。 influence high prices (for non-intervention group) |  |  |
|  |  |  |
