# Supplementary material for: Modelling enablers and barriers to improve uptake of a fruit and vegetable voucher scheme (the fresh street community) in England: a TISM-MICMAC approach
Source: BMC Public Health. 2026 Mar 21;26:1399. doi: 10.1186/s12889-026-27063-3 (PMC13126962; doi:10.1186/s12889-026-27063-3)
Supplement: Supplementary file 3 — Supplementary Material 3. [file 12889_2026_27063_MOESM3_ESM.docx]

Appendix A

**Table A1. Initial reachability matrix of Plymouth**


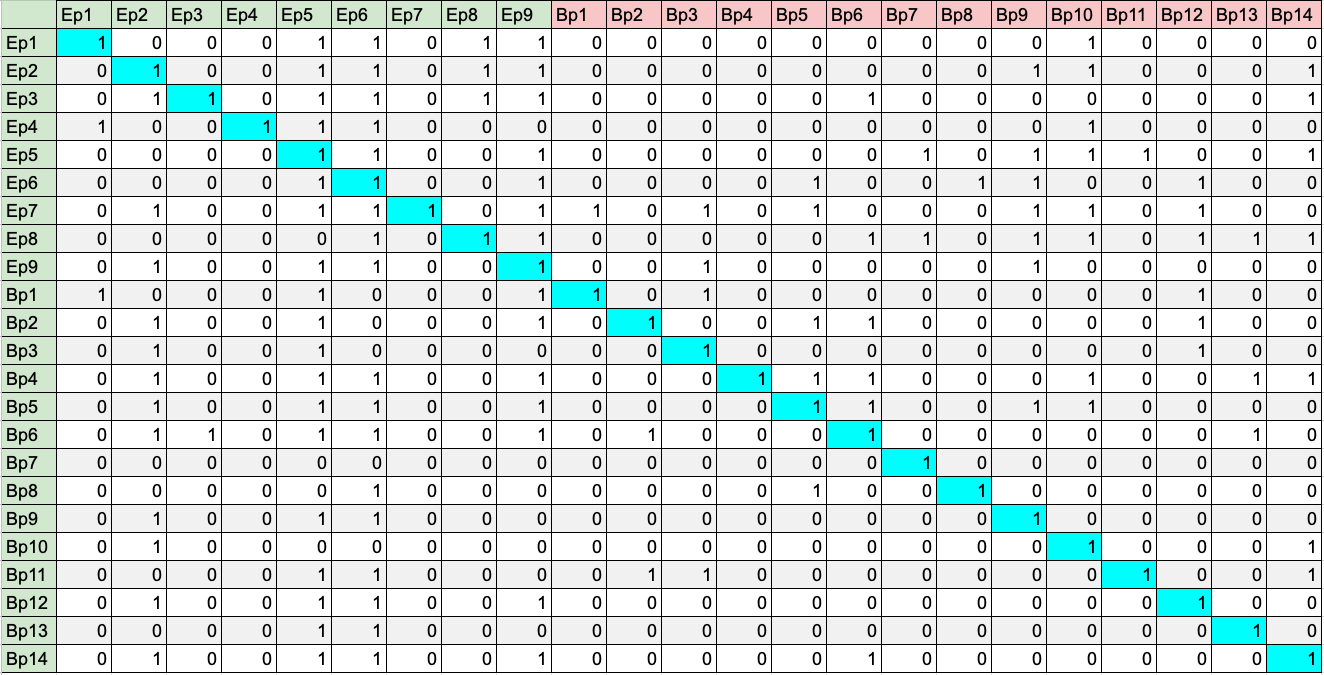


**Table A2. Initial reachability matrix of Reading**


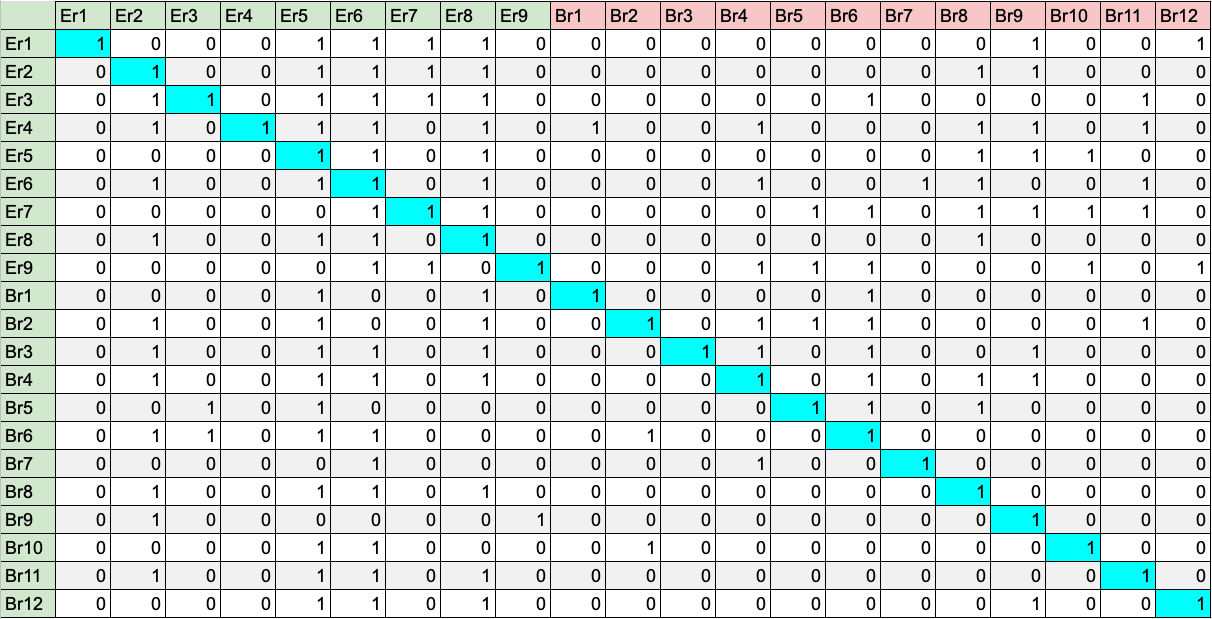


Appendix B

**Table B1. Final reachability matrix of Plymouth**

**
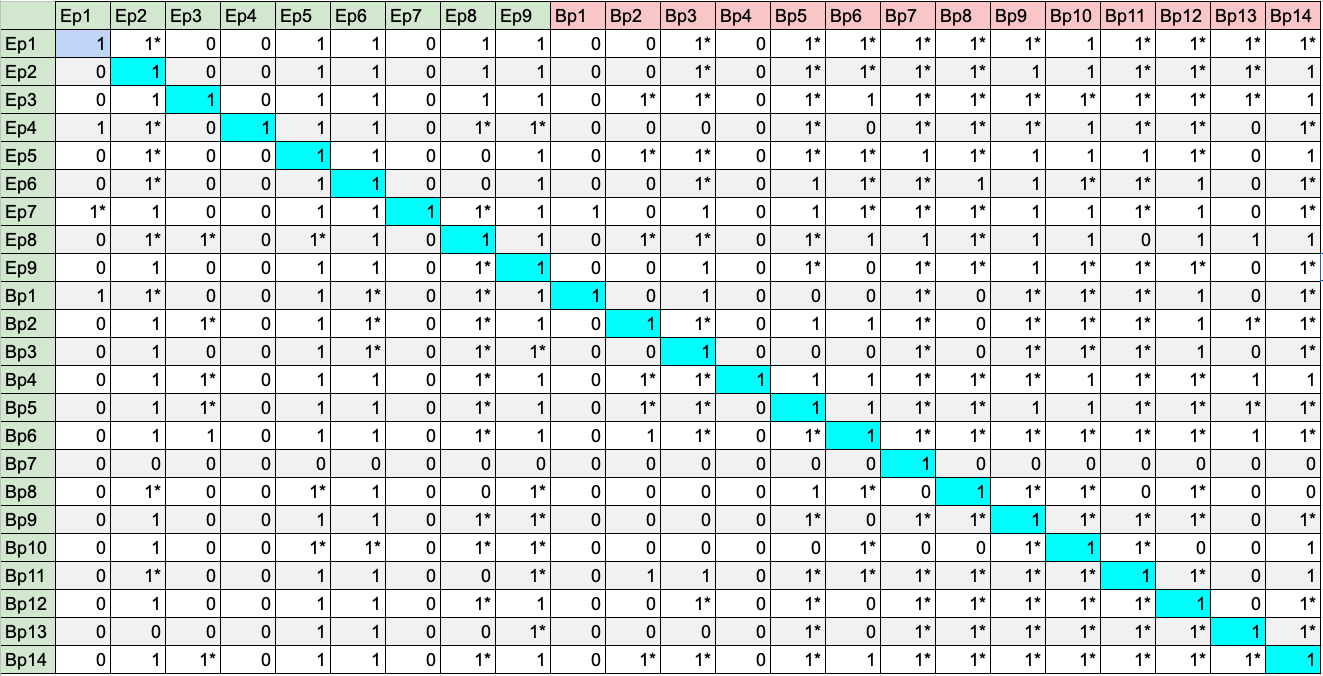
**

**Table B2. Final reachability matrix of Reading**

**
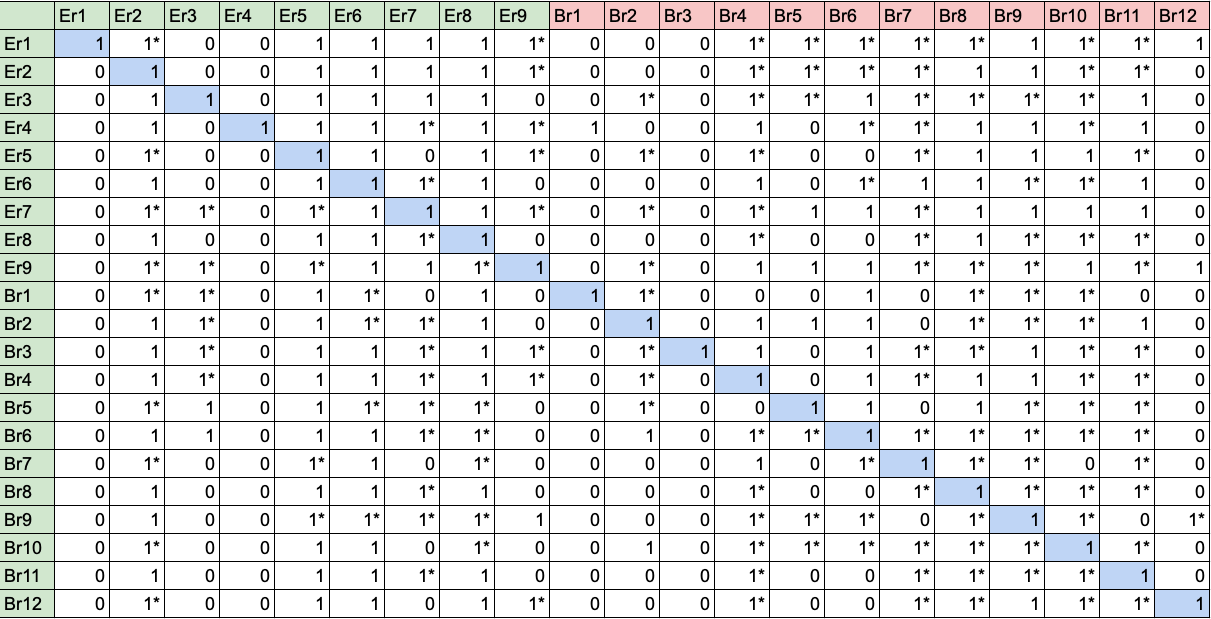
**

Appendix C

**Table C1. Level partitioning of Plymouth**


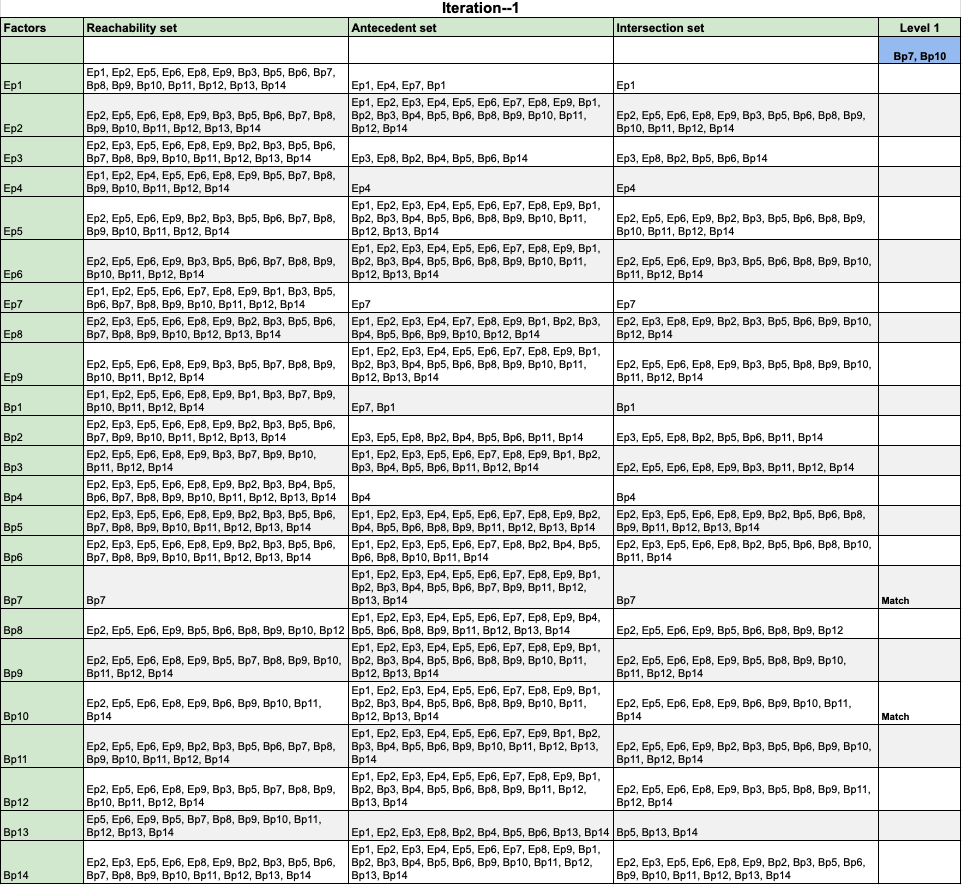


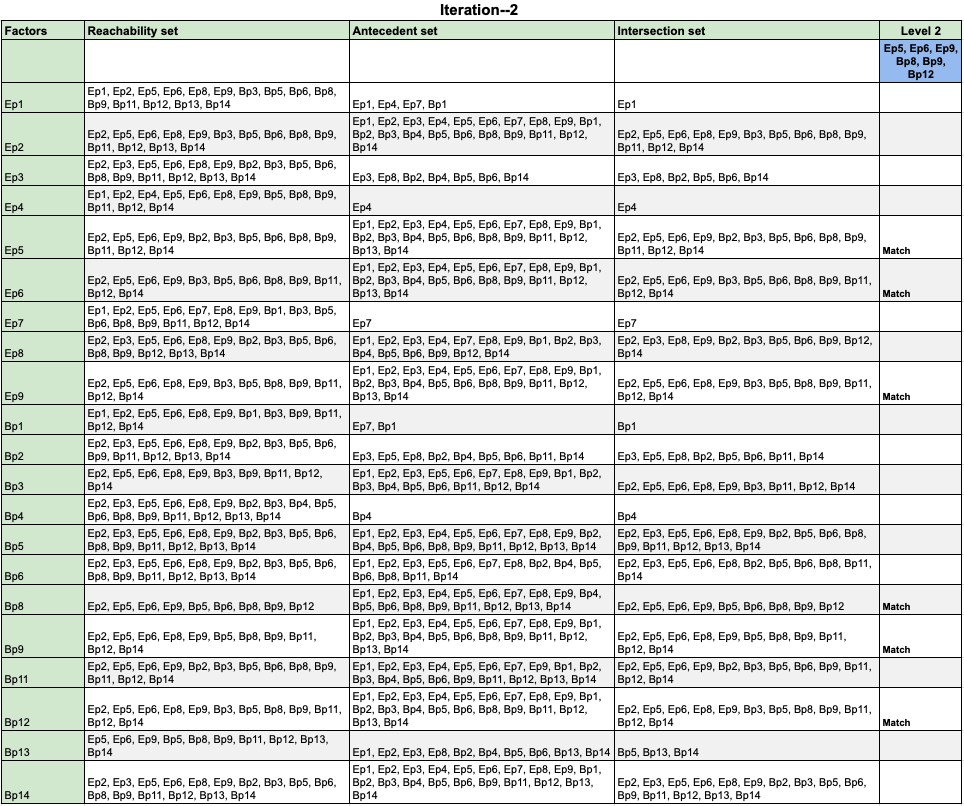


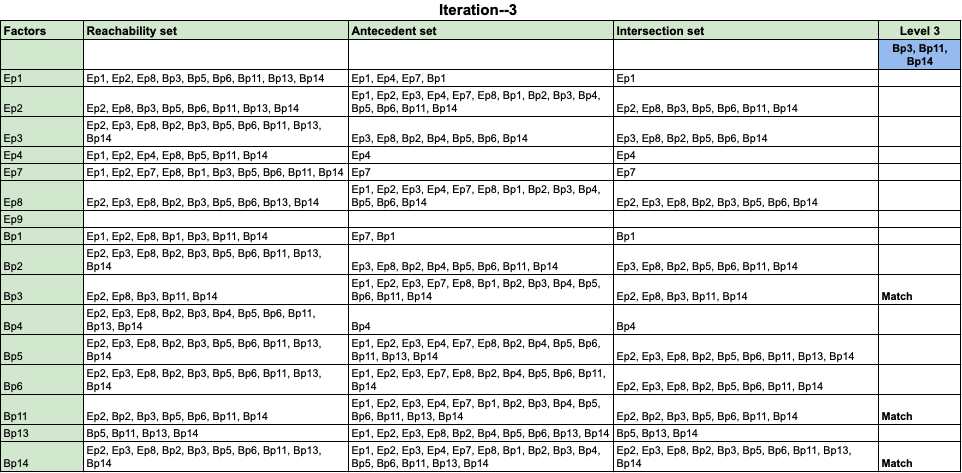


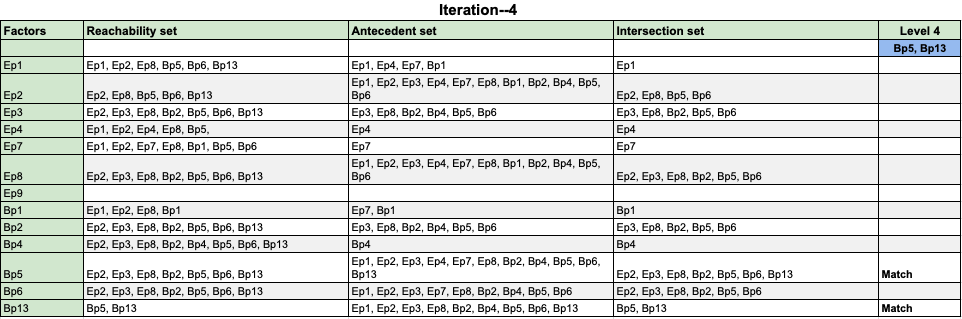


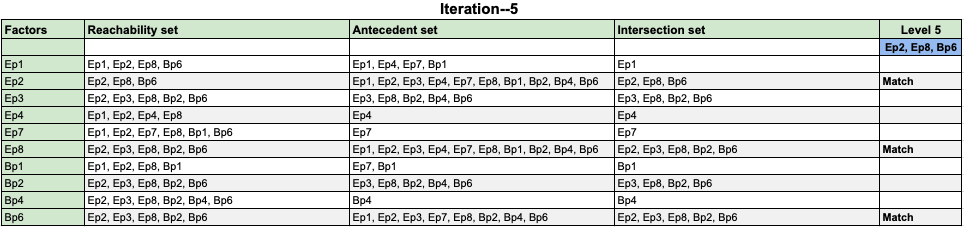


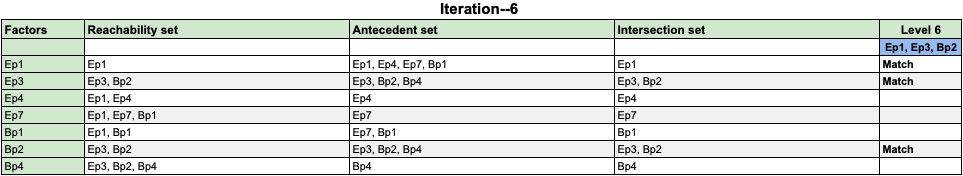


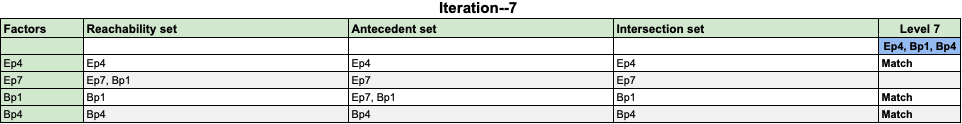


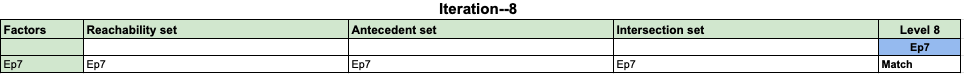


**Table C2. Level partitioning of Reading**


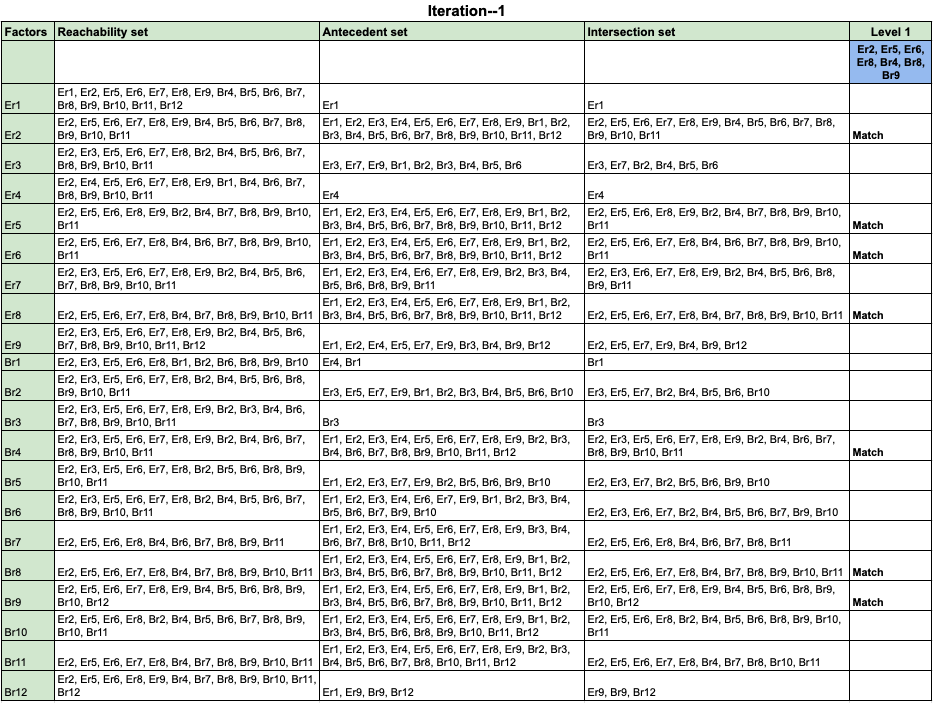


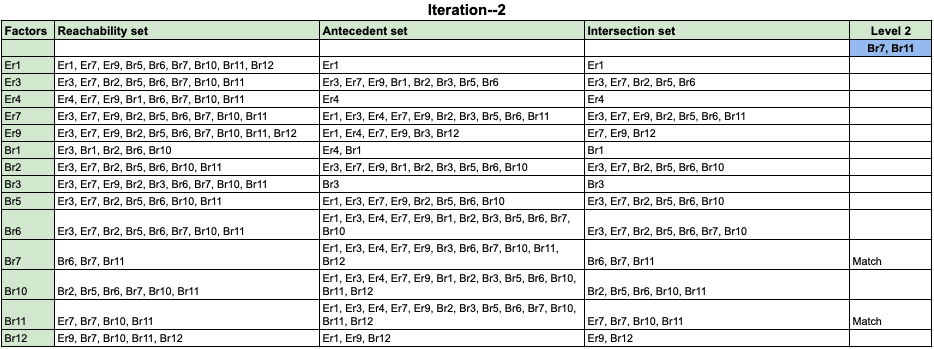


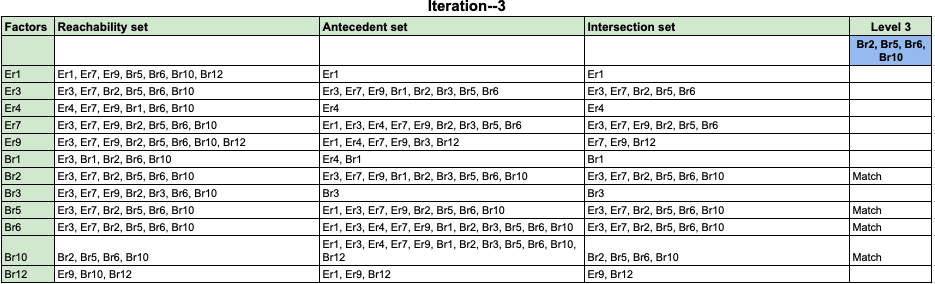


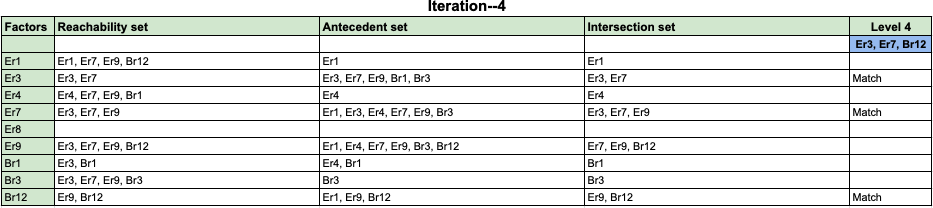


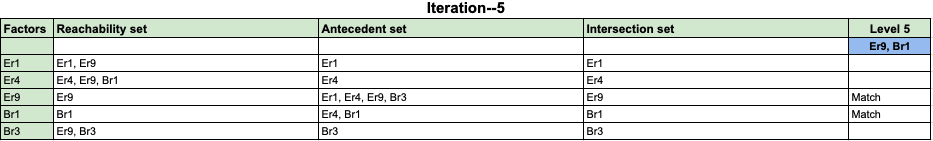


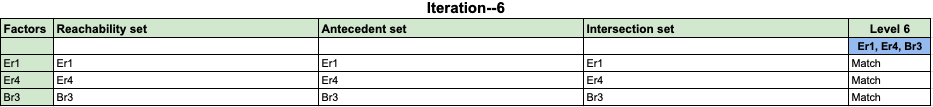


Appendix D

**Table D1. Interpretation statements of Plymouth**

| **Relation** | **Interpretation** |
| --- | --- |
| Ep7---Ep2 | A convenient location minimises travel time and costs, making it easier for households to access vouchers and benefits, which in turn maximises the impact of direct financial support. |
| Ep7---Ep5 | It facilitates access, strengthening community engagement. |
| Ep7---Ep6 | It increases visibility, giving residents more chances to interact and share positive experiences--thereby strengthening community bonds and driving word of mouth promotion. |
| Ep7---Ep9 | Convenient location makes participation easier to integrate into routines. |
| Ep7---Bp3 | Since it's close, can mitigate the inconvenience. |
| Ep7---Bp5 | Visibility and accessibility of the stall reduces low awareness. |
| Ep7---Bp9 | Avoiding community centres reduces stigma of being disadvantaged |
| Ep7---Bp12 | A convenient location reduces travel challenges, making it easier for individuals with long-term illnesses or specific dietary needs to access fresh produce independently, thereby lessening reliance on others. Potentially, when locals use the market stall on behalf of voucher holders, they are more likely to support those in need if it remains local. |
| Bp4---Ep2 | Directly affect funding. |
| Bp4---Ep5 | A short project duration limits the time available for building trust, consistent interactions and engagement, which are essential for developing strong social connections and delivering lasting educational impact. |
| Bp4---Ep6 | Building relationships and trust in a community hub takes time, so when the project ends, it can negatively impact local trust as the valuable connections that were built are lost. |
| Bp4---Ep9 | It prevents long-term habit formation. |
| Bp4---Bp5 | A short project duration limits the time for outreach and promotion, making it harder for information to spread widely, which results in lower awareness among the community |
| Bp4---Bp10 | Peoples shopping behaviours and spending will change after project ends. When the project ends, it makes life harder for those who rely on it. |
| Bp4---Bp14 | Insufficient habit formation limited educational impact and temporary behaviour change. |
| Bp1---Ep1 | Bad weather damage produces or delay deliveries, reducing freshness. |
| Bp1---Ep5 | Rain can reduce footfall, and people may not hang around to chat. |
| Ep4---Ep1 | It ensures freshness and quality. |
| Ep4---Ep5 | Using local and seasonal produce encourages shared learning about where their food comes from, how to cook it, and the benefits of eating seasonally. It helps to strengthen social connections. |
| Ep4---Bp10 | The price may be lower compared to other supermarkets. |
| Ep3---Ep2 | It is the mechanism through which financial support is delivered. Due to its ease of use, it attracted many people to participate in the voucher scheme which provides free fruits and vegetables. |
| Ep3---Ep5 | It gives people sometimes to talk to each other or even share vouchers with others. |
| Ep3---Ep8 | Vouchers are in £1 and £2s so people have a choice of how much to spend. Also, vouchers can be saved and spend in one go. |
| Ep3---Bp6 | The vouchers take a long time for staff to scan, as they are in small denominations. Time is also required to hand them out. |
| Ep1---Ep6 | People attend because they are happy with the produce, they let others know and spread the word. |
| Ep1---Ep9 | Enhance, People wouldn’t come repeatedly if the produce was poor quality. |
| Bp2---Ep2 | Some may not be able to attend and benefit from the financial support. |
| Bp2---Ep5 | Limited opening times lead to limited social connection. |
| Bp2---Ep9 | Limited opening hours make it difficult for many working families to attend. |
| Bp2---Bp5 | Limited opening times reduce awareness of the project; extending them would allow more people to visit, thereby increasing visibility. |
| Bp2---Bp12 | It may negatively affect those shopping on behalf of people suffering from long-term illness, as the order-and-pickup event operates for only two hours every fortnight, and the market stall is open just once a month. |
| Ep2---Ep6 | It helps build trust and goodwill within the community by addressing real needs. People are more likely to share their positive experiences with others, spreading awareness through word of mouth. |
| Ep2---Ep9 | It encourages regular use of the market stall, as people get free voucher to use. Over time, this consistent pattern helps establish a new life routine. |
| Ep2---Bp9 | It may unintentionally create stigma among recipients and non-recipients. |
| Ep2---Bp10 | It directly reduces cost pressures for participants. |
| Ep2---Bp14 | It allows people to choose fruits and vegetables without worrying about cost, giving them the freedom to explore and select their preferred items. |
| Ep8---Ep6 | At the market stall, people can choose the produce and quantity they want, which enhances their sense of autonomy and satisfaction. This makes them more likely to have positive experiences and share them with others. |
| Ep8---Bp6 | Setting up a monthly market stall adds to the workload for the research team and CFRs, including ordering, stock management, and operating the till. |
| Ep8---Bp7 | People living alone usually don't eat as much as those with families. At the market stall, they can choose exactly what they need and want, helping them reduce waste and shop more efficiently. |
| Ep8---Bp9 | At the market, people can choose what they want instead of having selections made for them, which helps reduce feelings of stigma. |
| Ep8---Bp10 | People can have control over how much can afford to spend, and can make decisions on what to buy based on the energy costs required to cook. |
| Ep8---Bp12 | People with dietary restrictions can choose the produce that suits them. |
| Ep8---Bp13 | It increases workload and challenge for research team and CFRs operating the till. |
| Ep8---Bp14 | Choice allows people to select FVs that match their preferences, increasing satisfaction and use. |
| Bp6---Ep2 | Negative impact, not enough staff to provide services (financial support). |
| Bp6---Ep3 | Administering the vouchers to participants and scanning vouchers creates significant workload. |
| Bp6---Ep6 | The project needs enough CR/staff resource to work in pairs to conduct door knocking. |
| Bp6---Bp2 | With more resource more hours may have been possible. |
| Bp6---Bp13 | There aren’t enough time or resources to train the research team or hire professionals to run the market stall efficiently. |
| Bp5---Ep2 | Low/lack of awareness reduces householder to use the stall and vouchers and reduces data collection opportunities/data points. |
| Bp5---Ep5 | Low awareness of the project limits participation, reducing opportunities for social interaction and educational engagement around food and healthy habits. |
| Bp5---Ep6 | Fewer people know about the initiative, reducing opportunities for organic interactions and trust-building. Without awareness, community members cannot engage, share experiences, or advocate for the project. |
| Bp5---Bp9 | Low awareness of the project can increase stigma, as people may misunderstand why some receive vouchers and others don't, leading to feelings of exclusion or unfairness. |
| Bp5---Bp10 | Some missed out on the financial support available due to lack of awareness of the project. |
| Bp13---Ep6 | It may reduce participant satisfaction and limit positive word of mouth, ultimately weakening community relationships. |
| Bp3---Ep2 | It's difficult to attend for many working families. |
| Bp3---Ep5 | It encourages people to meet more often (e.g., twice in the same week). |
| Bp3---Bp12 | The order and collection processes take two days, making it even more difficult to attend. |
| Bp11---Bp3 | Many people don't have time to attend. |
| Bp11---Bp14 | Lack of time for cooking can limit people’s ability to prepare certain FVs, leading them to prefer processed foods that are quicker and easier to make. |
| Bp14---Ep2 | It may reduce uptake of the scheme, hence limiting the number of people who can benefit from the free vouchers. |
| Bp14---Ep5 | It can reduce engagement and limit opportunities for shared learning, cooking discussions, and connection with others around healthy eating. |
| Bp14---Ep9 | It's unlikely for people to build life routine to attend the events if they don't like FVs. |
| Ep6---Ep5 | It fosters trust and word-of-mouth promotion. |
| Ep6---Bp5 | It directly combats low awareness. |
| Ep6---Bp8 | Door to door outreach helps provide information verbally. It acts as a replacement for the letters distributed to project householders. |
| Ep6---Bp9 | It reduces stigma by normalising participation. |
| Ep5---Ep6 | Strengthen word-of-mouth and trust. |
| Ep5---Ep9 | Engagement activities make for a good atmosphere, people want to attend regularly to shop and talk. |
| Ep5---Bp7 | Reducing isolation, practical skill-sharing, overcoming reluctance to cook. |
| Ep5---Bp9 | Social connection and education about the project may reduce any stigma associated with being involved. |
| Ep5---Bp10 | Engagement activities can give ideas for money saving tips, quick cook/no cook recipes. |
| Ep5---Bp11 | Engagement activities can give ideas for money saving tips, quick cook/no cook recipes. |
| Bp12---Ep5 | It limits participation in the engagement activities, reducing opportunities for learning and bonding over food. |
| Bp12---Ep9 | Unable to access market stall directly or indirectly to build life routine. |
| Ep9---Ep2 | Voucher holders may adapt their routine to benefit from being able to use free vouchers. |
| Ep9---Ep5 | People come regularly increase individuals’ social connectivity. |
| Ep9---Ep6 | It builds familiarity, enhancing community advocacy. |
| Ep9---Bp3 | When the scheme becomes part of participants’ routines (e.g., marking pickup dates on calendars), familiarity reduces perceived inconvenience. Regulars adapt to the two-day process (order & pickup), normalising it over time. |
| Ep9---Bp9 | As it becomes part of people’s regular routine, any stigma reduces. |
| Bp9---Ep2 | It may negatively impact householders by discouraging them from using the voucher scheme or making them feel stigmatised for not being selected. |
| Bp9---Ep5 | Perceived stigma discourages householder engagement with the free voucher scheme. |
| Bp9---Ep6 | Undermining trust and participation, silencing advocacy, amplifying misconceptions. |
| Bp8---Ep6 | It leads to low awareness and hinders understanding of communications. |
| Bp8---Bp5 | It directly causes low awareness of the project as people don't read the information letters. |
| Bp10---Ep2 | The crisis heightens reliance on direct financial support, increasing participation and reinforcing the project’s role as a crisis buffer. |
| Bp10---Bp14 | With limited budgets, people tend to buy cheap processed food rather than fresh produce. |

**Table D2. Interpretation statements of Reading**

| **Relation** | **Interpretation** |
| --- | --- |
| Er1---Er6 | People attend because they are happy with the produce, they let others know and spread the word. |
| Er1---Er8 | People wouldn’t come repeatedly if the produce was poor quality. |
| Er1---Br12 | The high quality influences the higher prices. Better quality fruit and veg may demand a higher premium. |
| Er4---Er2 | A convenient location minimises travel time and costs, making it easier for households to access vouchers and benefits, which in turn maximises the impact of direct financial support. |
| Er4---Er5 | A convenient location draws more community members, making it easier for them to attend social and educational activities, which in turn fosters stronger interpersonal connections and shared learning experiences. |
| Er4---Er6 | Stall is visible so promote positive word-of-mouth within the neighbourhood. |
| Er4---Er8 | Householders may be likely to change their behaviours and use the market stall regularly if it is convenient. |
| Er4---Br1 | Convenient location diminishes the negative impact of bad weather. |
| Er4---Br4 | Visibility of the stall reduces low awareness. |
| Er4---Br8 | Some feel less stigma as the stall is outside. |
| Er4---Br9 | If householders experience the advantage of reduced travel — for example, avoiding bus or taxi fares to other shopping outlets — they may find that, although produce at the stall is more expensive, it could still be cheaper overall than paying for transport. |
| Er4---Br11 | A convenient location makes it easier for those with long-term illnesses or dietary needs to access fresh produce independently and encourages locals to support voucher holders if the stall stays nearby. |
| Br3---Er2 | People may come to rely on the financial support and struggle when it ends. |
| Br3---Er5 | It reduced connectivity when market stall ceases to trade. |
| Br3---Er6 | Building relationships and trust in a community hub takes time, so when the project ends, it can negatively impact local trust as the valuable connections that were built are lost. |
| Br3---Er8 | People’s routines will need to adjust when project ends. |
| Br3---Br4 | It takes time to build awareness, so shorter project leads to lower awareness. |
| Br3---Br9 | Peoples shopping behaviours and spending will change after project ends. When the project ends, it makes life harder for those who rely on it. |
| Er9---Er6 | People form a good opinion of the stall, community centre and supplier. |
| Er9---Er7 | A good variety of FVs enhances the choice of produce available. |
| Er9---Br5 | It may increase time taken to make selections, increase the queuing time. |
| Er9---Br12 | The stall offers a good variety of foreign FVs, which drives up the cost. |
| Br1---Er5 | Rain can reduce footfall, and people may not hang around to chat. |
| Br12---Er5 | Householders stop or reduce using the stall as prices are higher. |
| Br12---Er6 | High prices negatively effect the word of mouth among the community. |
| Br12---Er8 | Householders stop or reduce using the stall due to higher prices, which negatively affects the development of life routine. |
| Br12---Br9 | It worsens the impact of the cost of living crisis, as people may not afford the produce without vouchers. |
| Er7---Er6 | Allowing people to choose the produce they want enhances their sense of autonomy and satisfaction. They are more likely to have positive experiences and share them with others. |
| Er7---Br6 | Ordering large quantities and variety of choice, estimating stock requirements and returning unsold produce adds to workload. Staff required to weigh produce and work at the till. |
| Er7---Br9 | Householders can have control over how much can afford to spend, and can make decisions on what to buy based on the energy costs required to cook. |
| Er7---Br11 | People with dietary restrictions can choose the produce that suits them. |
| Er3---Er2 | An easy-to-use voucher system streamlines the redemption process, ensuring that households quickly benefit from direct financial support by reducing barriers to accessing produce and saving money for other expenses. |
| Er3---Er5 | It gives people sometimes to talk to each other or even share vouchers with others. |
| Er3---Er7 | Vouchers are in £1 and £2s so people have a choice of how much to spend. |
| Er3---Br6 | The vouchers take a long time for staff to scan, as they are in small denominations. Time is also required to hand them out. |
| Br2---Er2 | Some people may not be able to attend and benefit from the financial support. |
| Br2---Er5 | Less available time for social connectivity at the stall/community hub. |
| Br2---Er8 | Limited opening hours on Saturday mornings may be limited to some householders so influence their behaviour to attend the stall or not. |
| Br2---Br4 | More frequent opening times would lead to greater awareness of the scheme, as people pass by. |
| Br2---Br5 | It can get quite busy and long queues, as the stall is only open two hours every week. |
| Br2---Br11 | It might negatively influence those shopping for others as stall isn’t open for long and limited to Saturday morning. |
| Br5---Br6 | It increases the workload. Several staff are necessary for the process. |
| Br5---Br8 | Embarrassment if unsure about how much have spent or if have enough vouchers before weighing FV. |
| Br6---Er2 | It has negative impact on direct financial support. As there isn't enough staff, the stall can only open on Saturdays. |
| Br6---Er3 | Administering the vouchers to participants and scanning vouchers creates significant workload to the community hub team. |
| Br6---Er6 | The project needs enough CFR/staff resource to work in pairs to conduct door knocking. Staff and volunteers work during the week and running and promoting the scheme on Saturday, making the workload too demanding. |
| Br6---Br2 | With more resource more hours may have been possible. |
| Br10---Er5 | People who lack time to cook also struggle to attend and engage with the scheme. |
| Br10---Er6 | Don't have time to attend and engage. |
| Br7---Er6 | It contributes to low awareness, and those who don’t speak English may struggle to understand the communications. |
| Br7---Br4 | Unable to comprehend information provided verbally or in writing about the project or just don't read the information letter. |
| Br11---Er2 | May not be able to access the community centre to collect vouchers/use stall to make use of the financial support. |
| Er8---Er2 | Voucher holders may adapt their routine to benefit from being able to use vouchers at the market stall. |
| Er8---Er5 | People come regularly increase individuals’ social connectivity. |
| Er8---Er6 | People come regularly as have formed good relationships with the team and help spread the word about the scheme. |
| Er6---Br4 | Doorstep interactions by community researchers will increase awareness of the project. Word of mouth information decreases low awareness. |
| Er6---Br7 | Door to door outreach helps provide information verbally. It acts as a replacement for the letters distributed to project householders. |
| Er6---Br8 | Trust built may reduce any stigma associated with being involved. |
| Er5---Er6 | Social connection and education foster engaging experiences that encourage conversation, trust, and shared learning, leading to stronger community ties and increased word-of-mouth promotion. |
| Er5---Er8 | The stall and engagement activities make for a good atmosphere, people want to attend regularly to shop and talk. |
| Br9---Er9 | During consultations between the community hub and the wholesaler, the produce range was reduced because they considered certain items too expensive for the stall's householders. |
| Br4---Er2 | Low/lack of awareness reduces householder to use the stall and vouchers and reduces data collection opportunities/data points. |
| Br4---Er5 | It reduces awareness of stall and hence participation in the project. |
| Br4---Er6 | People do not attend and talk about it. |
| Br4---Br8 | Limited awareness of the project’s objectives increases stigma and reduces the potential use of vouchers and the stall. |
| Br4---Br9 | Some missed out on the financial support available due to lack of awareness of the project. |
| Br8---Er2 | Stigma negatively affects householders’ willingness to use the voucher scheme or makes them feel excluded if they haven't been selected. |
| Br8---Er5 | Lack of engagement in the voucher scheme if householders feel stigmatised to use free voucher or community hub. |
| Br8---Er6 | Undermining trust and participation, silencing advocacy, amplifying misconceptions. |
| Br8---Er8 | When people don’t attend, it becomes difficult to establish a consistent routine. |
| Er2---Er6 | People talk to each other about the scheme. |
| Er2---Er8 | Receiving or collecting vouchers weekly becomes part of the routine. Collecting vouchers requires people to attend and can do so on same day as stall. |
| Er2---Br8 | Some feel they do not need the support and feel patronised, others feel it’s unfair they are not receiving support. |
| Er2---Br9 | The support helps people’s money go further. |

Appendix E

**Table E1. MICMAC binary matrix of Plymouth**

**
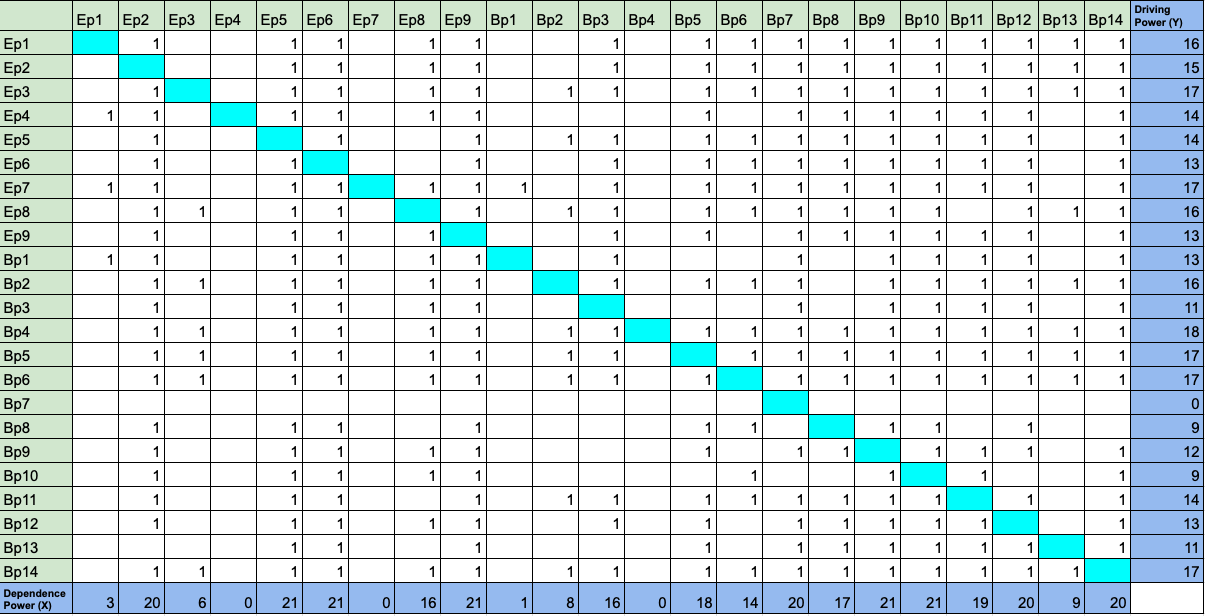
**

**Table E2. MICMAC binary matrix of Reading**

**
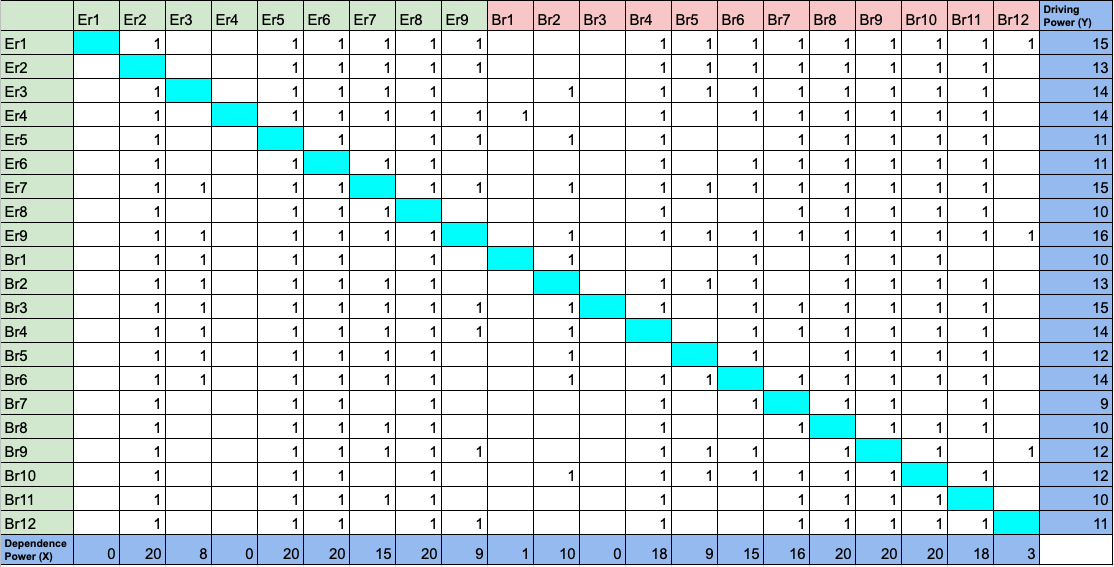
**
